# Multiparametric Bioresorbable Sensor for Doxorubicin Detection via Molecularly Imprinted Synthetic Receptors

**DOI:** 10.1002/advs.202524369

**Published:** 2026-03-03

**Authors:** Martina Corsi, Tiziano Di Giulio, Eleonora Vandini, Muhammad Ibrar Asif, Eleonora Daini, Antonietta Vilella, Giuseppina Leo, Alessandra Ottani, Cosimino Malitesta, Daniela Giuliani, Elisabetta Mazzotta, Giuseppe Barillaro

**Affiliations:** ^1^ Department of Information Engineering University of Pisa Pisa Italy; ^2^ Laboratory of Analytical Chemistry Department of Biological and Environmental Sciences and Technologies (Di.S.Te.B.A.) University of Salento Lecce Italy; ^3^ Department of Biomedical Metabolic and Neural Sciences University of Modena and Reggio Emilia Modena Italy

**Keywords:** bioresorbable sensor, doxorubicin monitoring, implantable diagnostics, molecularly imprinted polymer, multiparametric sensing

## Abstract

Monitoring chemotherapeutic drug concentrations directly at the tumor site remains a critical unmet need in oncology, as conventional pharmacokinetic assessments based on systemic circulation fail to capture the spatial and temporal heterogeneity of drug distribution within solid tumors. Here, we report a bioresorbable, multiparametric optical sensor designed for the in situ detection of the chemotherapeutic agent doxorubicin. The sensor integrates a nanostructured porous silica scaffold with a molecularly imprinted polymer (MIP) synthetic receptor that provides shape‐ and chemistry‐selective recognition of doxorubicin molecules. Molecular binding events are transduced through two orthogonal optical signals: i) shifts in effective optical thickness and ii) fluorescence intensity changes, enabling accurate and self‐validating quantification across clinically relevant concentration ranges. The sensor operates reliably in serum with a limit of detection as low as 0.1 µg/mL, and exhibits reversible performance with minimal signal drift (<15.3%) over 12 weeks —consistent with standard chemotherapy regimens. In vivo implantation studies in mice confirm biodegradation and biocompatibility, with no evidence of local or systemic toxicity. This platform introduces a versatile strategy for multiparametric, bioresorbable chemical sensing using MIP synthetic receptors, establishing a foundation for future implantable diagnostics in precision chemotherapy.

## Graphical abstract

A biodegradable molecularly imprinted polymer porous silica sensor enables dual optical readout of doxorubicin via fluorescence and effective optical thickness shifts. The combined transduction mechanisms provide enhanced reliability and quantitative accuracy over clinically relevant concentrations, representing a step toward transient implantable sensors for in vivo chemotherapeutic monitoring.

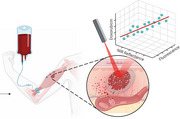

## 1 Introduction

Chemotherapy remains a cornerstone of cancer treatment, yet its success critically depends on maintaining optimal drug concentrations at the tumor site—an objective that remains elusive in clinical practice [1]. Despite advances in targeted therapies [2] and localized drug delivery systems [3, 4, 5, 6], pharmacokinetic assessment still relies predominantly on systemic markers, including plasma drug levels measured by liquid chromatography‐mass spectrometry (LC‐MS) techniques [7], organ toxicity indicators [8] (e.g., ALT, cardiac markers), and whole‐body imaging [9] (e.g. PET), all of which offer limited insight into the actual drug levels within tumor tissue. This disconnect is particularly problematic in solid tumors, where vascular heterogeneity, stromal barriers, and elevated interstitial pressure result in highly variable intratumoral drug distribution. Inadequate drug penetration into resistant tumor regions can lead to therapeutic failure, while off‐target accumulation may increase systemic toxicity [10]. Although systemic monitoring remains essential, localized approaches are increasingly recognized as necessary to complement systemic assessments and provide tumor‐specific pharmacokinetic insights.

Implantable sensors have recently been proposed to enable localized, in vivo drug monitoring with high spatial and temporal resolution [11, 12, 13]. Electrochemical [11, 13] and optical [12] sensors have shown promise in detecting small‐molecule chemotherapeutics, such as doxorubicin, in real time, typically relying on biological receptors (aptamers, DNA) and single‐mode transduction (electrochemical or fluorescence). However, these platforms remain constrained by the need for surgical retrieval, reliance on wired interfaces, and vulnerability to signal drift and biofouling, limiting their operational stability to only hours or days and hindering their long‐term use in vivo (Table S1).

Concurrently, the field of transient bioelectronics has made substantial progress in creating implantable, fully bioresorbable systems for monitoring physical and biochemical parameters [14, 15, 16] —such as temperature [17, 18], pressure [19, 20, 21], mechanical strain [19, 22, 23, 24], ions and biomolecules [18, 25, 26, 27, 28, 29, 30], —over clinically relevant timescales. These systems use biodegradable polymers and transient inorganic materials to construct soft, tissue‐conformal devices that safely degrade in vivo, eliminating the need for removal. Yet translating this approach to chemical sensing poses unique challenges: sensors must maintain high selectivity and operational stability under continuous exposure to biofluids while degrading predictably and safely [14]. Although bioresorbable sensors have been developed for ions (e.g., H^+^, Ca^2^^+^, O_2_) [18, 25, 26, 27, 28] and small biomolecules (e.g., glucose, dopamine) [26, 29, 30], fully degradable platforms for monitoring chemotherapeutic drugs are scarce. To date, doxorubicin detection with a bioresorbable sensor has only been demonstrated in a single study using human serum albumin (HSA) as the bioreceptor and fluorescence as a single‐mode transduction mechanism [31]. However, the use of a biological receptor limited the operational lifetime of the sensor to approximately one week—substantially shorter than the typical 12‐week duration of chemotherapeutic regimens in clinical practice.

Here, we introduce a multiparametric, fully bioresorbable optical sensor designed for localized monitoring of chemotherapeutic drug levels over a clinically relevant timescale of 12 weeks, building on previous studies of single‐mode, non‐bioresorbable MIP‐based sensors and biologically functionalized degradable optical platforms [27, 31, 32, 33, 34]. The sensor, only a few micrometers thick, is fabricated entirely from biodegradable materials and is conceived for localized implantation, such as within the tumor bed after surgical resection. It integrates a nanostructured porous silica scaffold with a molecularly imprinted polymer (MIP) synthetic receptor that provides doxorubicin‐specific recognition based on shape and chemical complementarity. MIPs serve as robust, synthetic alternatives to biological receptors, offering high chemical stability and the flexibility to be custom‐tailored to a broad range of target molecules [35]. Their integration into a biodegradable sensor platform, not yet explored so far, establishes a versatile strategy for molecular recognition that can be generalized to other chemotherapeutic agents or soluble biomarkers.

Critically, drug binding to MIP is transduced through two orthogonal optical outputs: i) shifts in effective optical thickness (EOT), reporting changes in local refractive index [36], and ii) changes in the fluorescence intensity of doxorubicin confined within the porous matrix. This dual‐mode architecture enables robust quantification over a broad concentration range of clinical relevance and allows internal validation of sensing events, enhancing measurement reliability in complex biological fluids. The sensor exhibits stable performance in serum, with a limit of detection (LoD) of 0.1 µg/mL, full reversibility, and sustained functionality for at least 12 weeks. Implantation studies in mice confirm biocompatibility and degradation of the device, with no evidence of local or systemic toxicity.

By enabling high‐resolution, spatiotemporal profiling of drug concentrations over 12 weeks in biologically relevant conditions, this platform establishes the foundation for future in vivo studies aimed at addressing a critical unmet need in cancer therapy. Its modular architecture and compatibility with synthetic receptor chemistry allow adaptation to a broad range of small‐molecule therapeutics and soluble biomarkers. More broadly, this work advances the emerging paradigm of bioresorbable chemical sensing by uniting selective molecular recognition, dual‐mode optical transduction, and complete in vivo degradation, defining a path toward intelligent, implantable diagnostic systems for adaptive, patient‐specific oncology.

## 2 Results and Discussion

**FIGURE 1:**
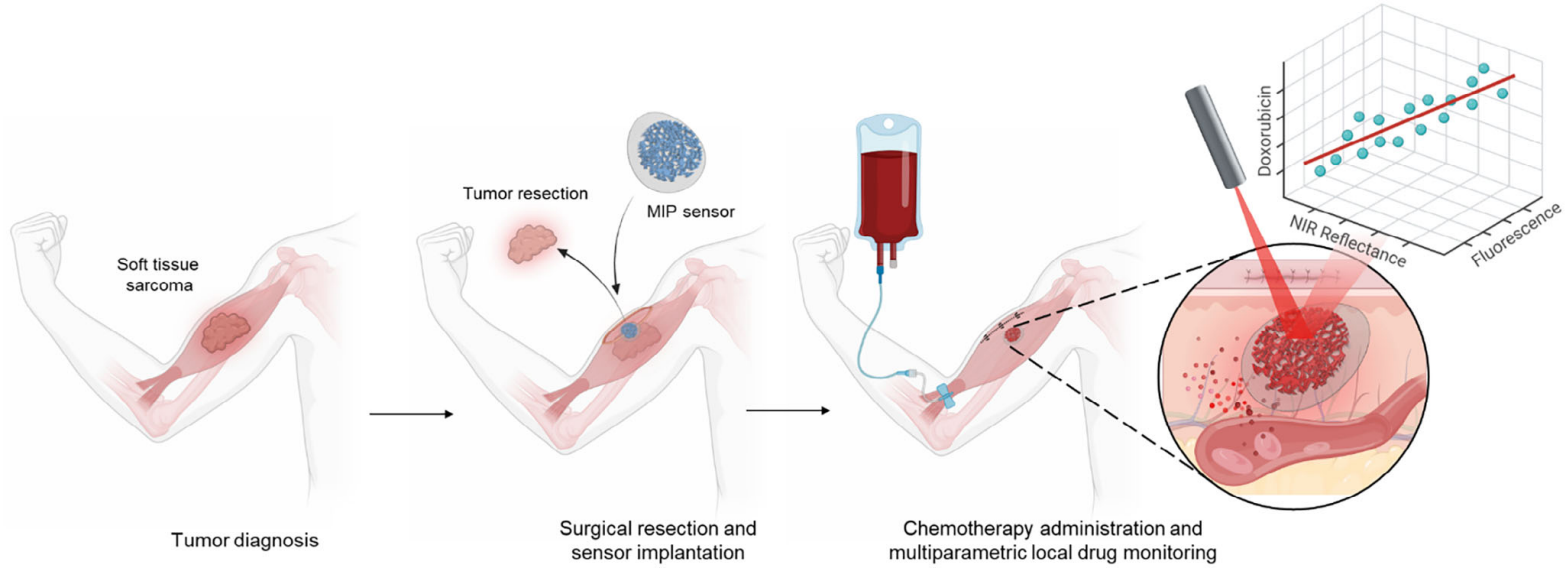
Conceptual illustration of in vivo multiparameter monitoring of doxorubicin using a MIP‐based biodegradable optical sensor. The schematic depicts the post‐surgical implantation of a multiparametric MIP‐based optical biosensor at the tumor resection site: 1) Tumor diagnosis: soft tissue sarcoma is identified in the arm. 2) Surgical resection and sensor implantation: after tumor removal, a fully biodegradable MIP sensor is implanted at the resection site to enable localized monitoring. 3) Chemotherapy administration and multiparametric monitoring: systemic doxorubicin is delivered intravenously, and the implanted sensor tracks local drug diffusion via two orthogonal optical readouts—effective optical thickness (EOT, NIR reflectance) and fluorescence intensity. These complementary signals ensure robust, high‐resolution monitoring of drug concentration dynamics at the tumor bed, supporting personalized and adaptive chemotherapy.

As illustrated in Figure 1, the sensor is designed for localized implantation within solid tumors following surgical resection, with the soft tissue sarcoma microenvironment presented as a representative model [37]. The sensor integrates a nanostructured porous silica membrane conformably coated with a molecularly imprinted polymer (MIP) recognition layer, enabling target‐specific molecular capture. Once clinically translated, the sensor is expected to enable continuous and selective monitoring of the chemotherapeutic agent doxorubicin as it diffuses from the vasculature into the tumor interstitial space. The sensor is intended to provide physicians with localized pharmacokinetic data directly at the implantation site for up to 12 weeks following chemotherapy, whether delivered as single agents or continuous infusions [37, 38, 39, 40]. The data, collected both in hospital settings and during patients’ daily activities at home when the sensor is paired with a wearable electronic patch [31], are aimed to complement systemic monitoring to guide dose adjustment once therapeutic indices are established, with the ultimate goal of enhancing treatment precision and reducing locoregional recurrence.

High‐resolution spatiotemporal profiling of the drug concentration is achieved via two orthogonal optical readouts: shifts in the reflectance spectrum and variations in fluorescence intensity. The MIP‐coated nanostructured silica membrane functions as a backscattering interferometer, exhibiting a peculiar reflectance spectrum that red shifts upon doxorubicin binding. This shift is quantified in the near‐infrared (NIR) region, where skin exhibits high optical transparency [41, 42, 43], by measuring the effective optical thickness (EOT = 2n·t) of the interferometric sensor, with n representing the effective refractive index and t the physical thickness of the sensor. In parallel, doxorubicin's intrinsic fluorescence [44] peaked at 580 nm serves as a complementary signal. Upon accumulation within the nanostructured silica matrix, its fluorescence emission is amplified up to tenfold and can be acquired through the skin [27, 31], providing a quantitative fluorescence response proportional to the local drug concentration. The combined use of reflectance and fluorescence measurements thus enables robust, real‐time tracking of pharmacokinetics within the tumor microenvironment, offering an integrated and highly sensitive approach to in vivo drug monitoring.

This transient sensing platform provides a clinically relevant strategy to support adaptive chemotherapy, enhancing therapeutic precision while minimizing systemic toxicity. Critically, the device is engineered to fully degrade into biocompatible byproducts after its functional lifetime, eliminating the need for retrieval. By directly capturing the local pharmacokinetics at the tumor site, this approach addresses a key challenge in oncology: the ability to quantify drug exposure in vivo with both temporal continuity and spatial specificity. Furthermore, the incorporation of MIP receptors affords molecular versatility, enabling the platform to be adapted for the detection of other oncologic drugs or soluble tumor biomarkers. This flexibility positions the sensor as a promising tool for next‐generation implantable diagnostics, capable of enhancing personalized cancer treatment across a range of therapeutic contexts.

**FIGURE 2:**
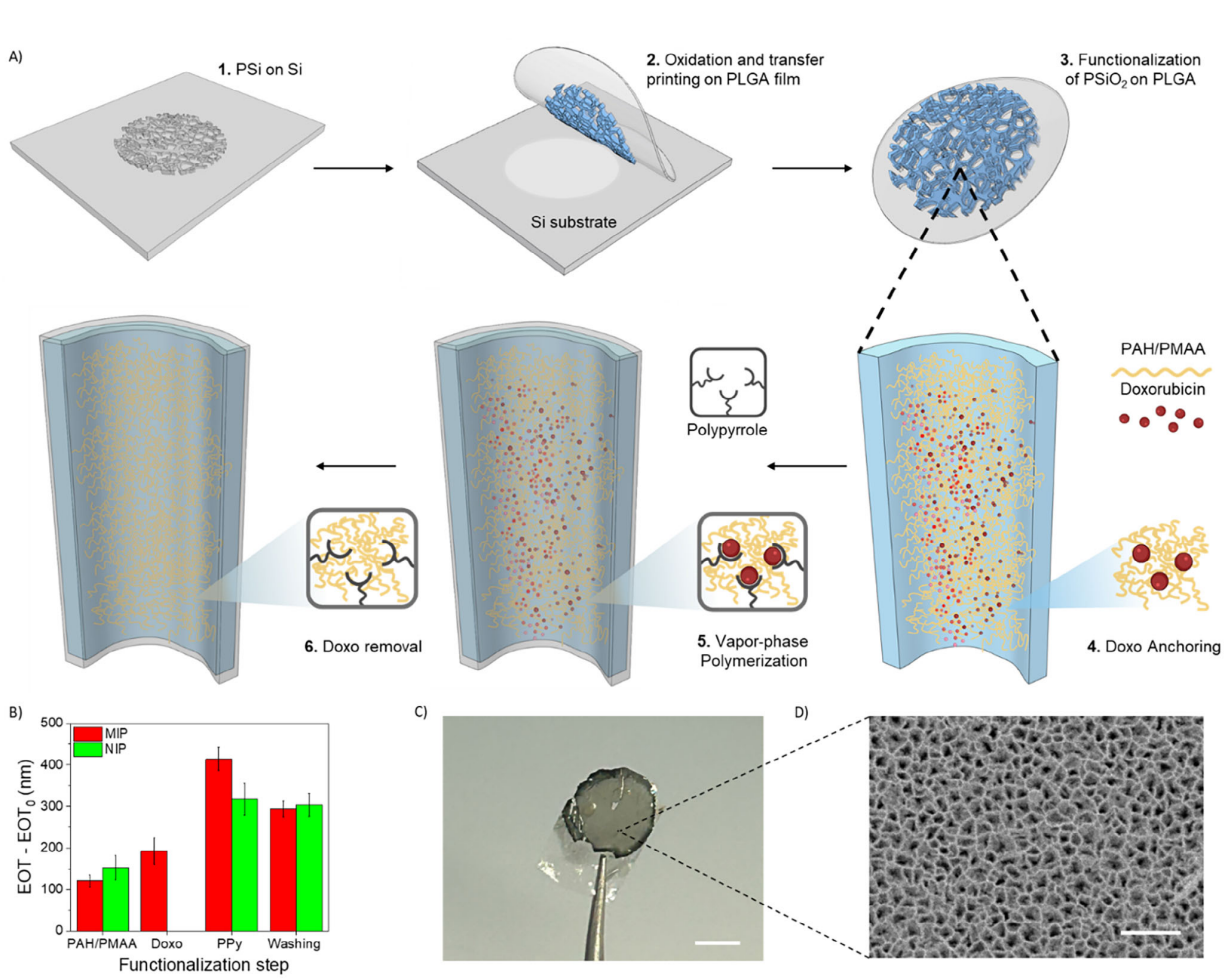
Fabrication and structural characterization of the MIP‐based multiparametric doxorubicin sensor. (A) Schematic overview of the sensor fabrication process: (1) fabrication of a porous silicon membrane; (2) thermal conversion to porous silica (PSiO_2_) followed by transfer printing onto a biodegradable PLGA substrate; (3) layer‐by‐layer (LbL) deposition of charged polyelectrolytes; (4) electrostatic anchoring of doxorubicin; (5) vapor‐phase polymerization of pyrrole to form a conformal polypyrrole (PPy) layer; (6) washing step to remove the doxorubicin template, resulting in the formation of molecularly imprinted recognition sites. (B) Quantitative tracking of fabrication steps using effective optical thickness (EOT) measurements. The EOT value after oxidation (i.e., EOT_0_) is used as a reference. Data are shown as mean ± SD, n = 3. (C) Optical image of the final MIP–PSiO_2_ sensor on the PLGA substrate. Scale bar: 500 µm. (D) Top‐view SEM image of the MIP–PSiO_2_ sensor, showing a uniform pore morphology with an average pore diameter of ∼20 nm. Scale bar: 250 nm.

The sensor is prepared by producing and transferring a micrometer‐thick (∼5 µm) nanostructured porous silica (PSiO_2_) membrane on a thin (∼50 µm) poly(lactic‐co‐glycolic acid) (PLGA) substrate (Figure 2A(1–3)), both selected for their biodegradability and biocompatibility [45, 46, 47]. The inner surface of the silica nanopores is functionalized via electrostatic layer‐by‐layer (LbL) [27, 48, 49, 50] deposition of nanometer‐thick polyelectrolytes, beginning with a cationic layer of poly(allylamine hydrochloride) (PAH), followed by an anionic layer of poly(methacrylic acid) (PMAA), ultimately yielding a net negatively charged surface. Doxorubicin is subsequently loaded through electrostatic interaction with the PMAA‐functionalized surface at pH 5.8 promoting its cationic form (pK_a1_ = 8.1) (Figure 2A (4)). To form the molecularly imprinted polymer (MIP), the architecture is then exposed to ferric chloride (FeCl_3_), which acts as an oxidizing agent during the next vapor‐phase polymerization of pyrrole, yielding a conformal, nanometer‐thin (∼2 nm) layer of polypyrrole (PPy) [34] (Figure 2A (5); Figure S2). This latter encapsulates the doxorubicin molecules during polymer growth without obstructing or altering the intrinsic pore architecture (Figure S2). Subsequent selective removal of doxorubicin generates molecularly imprinted cavities that are complementary in shape, size, and chemical functionality to the target molecule (Figure 2A (6)). It is important to emphasize that the LbL functionalization step promotes conformal, homogeneous loading of target molecules across the nanoporous scaffold, which significantly improves imprinting fidelity and, in turn, enhances both the reproducibility and magnitude of the sensor response.

The fabrication of the sensor was systematically monitored using reflectance spectroscopy, with changes in EOT serving as a quantitative metric to assess modifications within the porous interferometric structure. The deposition of polyelectrolytes induced a progressive increase in EOT, consistent with the red shift of the interference fringes due to conformal coating of the silica nanopores, as compared to the baseline value of the bare PSiO_2_ interferometer (Figure 2B; Figure S1A). Subsequent doxorubicin loading further increased the EOT, reflecting successful incorporation of the drug into the functionalized pores. This step was also associated with a decrease in reflectance intensity between 400–600 nm, attributable to the characteristic absorption band of doxorubicin near 480 nm [51] (Figure S1B). Further increase in EOT was observed following the vapor‐phase polymerization of pyrrole, confirming the deposition of a conformal PPy layer on the internal pore surface. The PPy coating also caused broadband attenuation in the reflectance spectrum due to its optical absorbance. Importantly, the formation of molecular recognition sites was assessed by comparing EOT values in molecularly imprinted and non‐imprinted polymer (NIP) structures after the final washing step. The selective removal of the doxorubicin template from the MIP resulted in a statistically significant decrease in EOT, attributable to the reduction in the average refractive index following the removal of the drug from the polymer film (Figure 2B). In contrast, the EOT of the NIP remained unchanged, as expected. Figure 2C displays the final MIP–PSiO_2_ sensor integrated onto the PLGA carrier substrate. The surface morphology of the sensor is shown in Figure 2D, revealing the nanostructured architecture with an average pore diameter of ∼20 nm and porosity of ∼51% (Figures S2 and S3).

**FIGURE 3:**
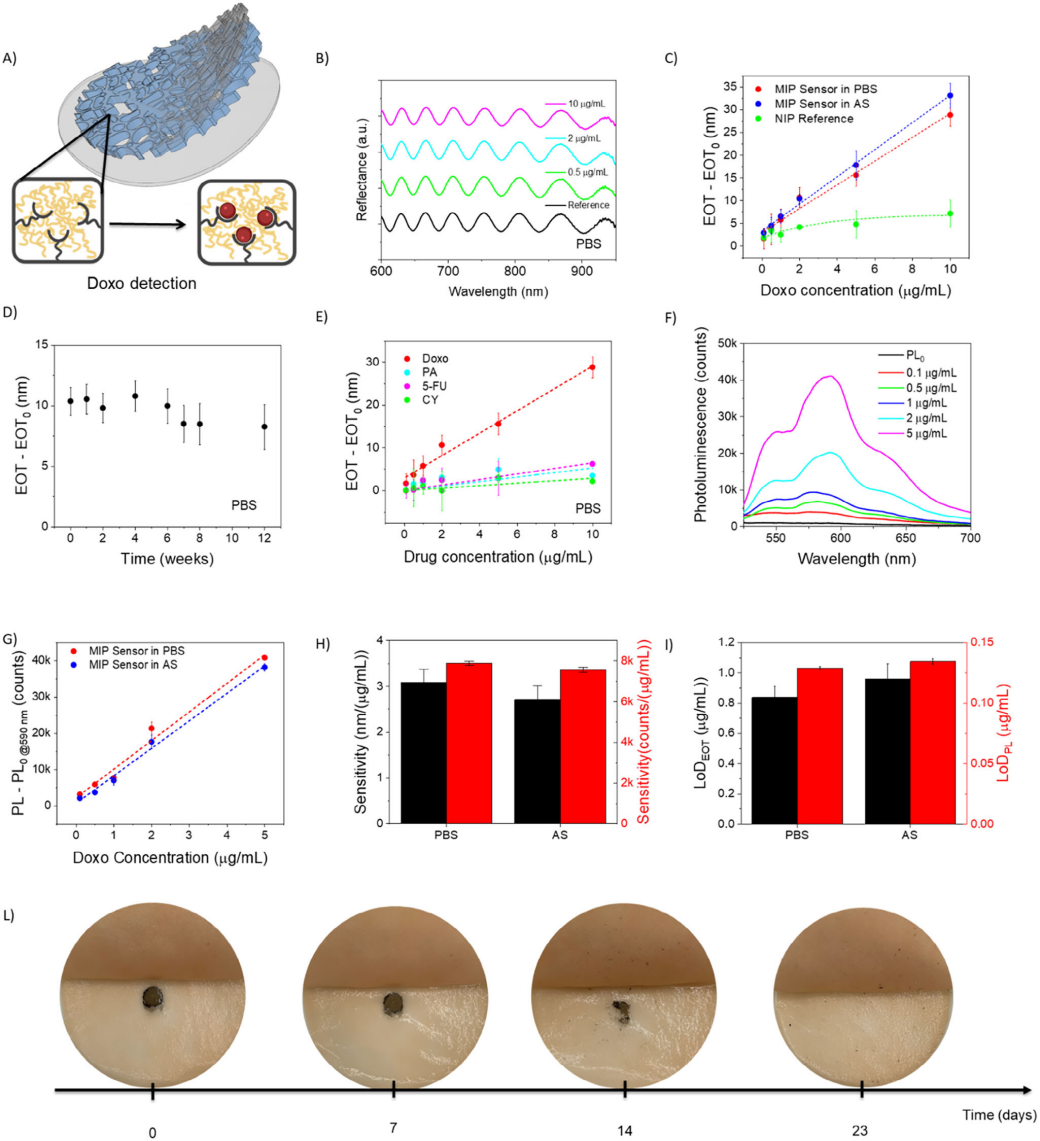
Multiparameter doxorubicin sensing. A) Sketch of doxorubicin interaction with PPy‐based MIP on PSiO_2_ scaffold. B) Reflectance spectra before (reference) and after incubation of doxorubicin. C) Calibration curves (EOT‐EOT_0_ versus doxorubicin concentration) recorded on MIP and not imprinted polymer (NIP) sensors in the range 0.1–10 µg/mL. EOT_0_ is measured in PBS and AS without doxorubicin and used as a reference. Dashed lines represent fits to the calibration curves. D) MIP sensor response (calculated as EOT‐EOT_0_) versus time measured over 12 weeks at doxorubicin concentration of 2 µg/mL. The EOT value before incubation (i.e., EOT_0_) is used as reference (n = 3). E) Selectivity results comparing the MIP sensor response to doxorubicin and to interfering molecules (PA,5‐FU, CY) at different concentrations from 0.1 to 10 µg/mL in PBS buffer. The EOT_0_ is measured in PBS without doxorubicin and interfering molecules and used as reference. Dashed lines represent linear fits to the calibration curves. F) photoluminescence (PL) spectra acquired from the MIP sensor after incubation in AS solution with different doxorubicin concentrations. PL_0_ is the signal recorded in AS without doxorubicin. G) Calibration curve of the MIP sensor, namely, PL intensity at 590 nm vs. doxorubicin concentration in PBS and AS. The EOT value before incubation (i.e., EOT_0_) is used as reference. Dashed lines represent linear fits to the calibration curves. H) Sensitivity of the MIP sensor in PBS and AS. I) LoD of the MIP sensor in PBS and AS. L) Accelerated degradation of the sensor under artificial skin at 45°C, showing progressive degradation over time. Data in (B–E, G–I) are shown as mean ± SD, n = 3.

The sensor was evaluated for multiparametric detection of doxorubicin across a concentration range of 0.1–10 µg/mL, encompassing levels typically found in tissue following intravenous administration [37, 39]. Detection was achieved by concurrently monitoring two orthogonal optical signals: changes in EOT, which reflect alterations in the refractive index of the porous interferometric structure in the NIR range upon doxorubicin binding, and variations in the intrinsic fluorescence intensity of doxorubicin molecules confined within the sensor matrix (Figure 3A).

The change in EOT (ΔEOT = EOT − EOT_0_) in the NIR window was measured against EOT_0_, which corresponds to the baseline value measured in buffer without doxorubicin. Reflectance spectra were recorded after 60 min of exposure to doxorubicin solutions (Figure 3B), based on prior binding kinetics studies at 5 µg/mL, which indicated equilibration under these conditions (Figure S4). As shown in Figure 3C, the MIP–PSiO_2_ sensor exhibited a linear response over the 0.1–10 µg/mL range, with good reproducibility (RSD = 8.6%) and a LoD of approximately 0.8 µg/mL in PBS. The calibration performance of the sensor was fully retained in artificial serum (AS), as compared to phosphate‐buffered saline (PBS), indicating a robust and reliable operation in a complex biofluid under physiologically relevant conditions (Figure 3C). In contrast, the non‐imprinted PSiO_2_ (NIP) interferometer used as the control displayed a markedly reduced response, with signal saturation occurring at concentrations above 1 µg/mL. Extending the dynamic range of the MIP sensor to 150 µg/mL the sensor shows a saturation tendency above 50 µg/mL and fitting the calibration curve to a Langmuir adsorption model over the extended concentration range yields an apparent binding constant of K_0_ ≈ 2.6 × 10^4^ m ^−^^1^, consistent with reported values for MIP systems targeting small‐molecule drugs [52, 53] (Figure S5).

The sensor demonstrated excellent operational stability, reversibility, and reliability. When exposed to 2 µg/mL of doxorubicin over a 12‐week period under physiological conditions, the signal drift remained below 15.3% on average (Figure 3D). Notably, the sensor exhibited fully reversible behavior, with signal levels returning to baseline in the presence of drug‐free buffer, and restoring upon re‐exposure to doxorubicin (Figure S6A). This reversibility enabled repeatable sensing cycles, with consistent signal recovery upon re‐exposure to doxorubicin. Moreover, the consistent response across multiple cycles highlights the sensor's robust and reproducible performance over time (Figure S6B).

The selectivity of the MIP–PSiO_2_ sensor was validated against structurally unrelated chemotherapeutics, including paclitaxel (PA), 5‐fluorouracil (5‐FU), and cyclophosphamide (CY). At the lowest tested concentration, the sensor's response to doxorubicin was up to 20‐fold greater than that observed for the interfering compounds, reducing to a factor about 5 at the higher concentrations (Figure 3E; Figure S7A,B). The high selectivity of the system originates from the molecularly imprinted recognition sites, which are specifically tailored to doxorubicin. To elucidate the interactions governing doxorubicin binding, molecular mechanics (MM) simulations were performed (Figure S8), allowing for the identification of key non‐covalent forces involved, namely, hydrophobic interactions, hydrogen bonding, and van der Waals forces. These interactions were quantitatively assessed through cumulative binding energy calculations (Table S2). The binding energy of doxorubicin was significantly more favorable than that of competing molecules, suggesting that this energetic advantage underpins the MIP selectivity. This effect is likely reinforced by the preservation of functional moieties from the monomer units during the polymerization process, which ensures effective template recognition.

The changes in doxorubicin fluorescence, which displays characteristic emission in the 550–700 nm range, were evaluated by measuring the peak intensity at 590 nm against the doxorubicin concentration in the range 0.1–5 µg/mL in PBS and AS. Notably, the emission spectra of doxorubicin entrapped in the MIP sensor retained their characteristic line shape and peak position typical of doxorubicin solutions (Figure 3F; Figure S9A,B). The fluorescence intensity is linearly proportional to the drug concentration, with high reproducibility across the whole range (RSD = 1.8%) (Figure 3G). Moreover, the molecular confinement within the nanostructured sensor not only increased the effective local binding of doxorubicin but also amplified its emission intensity above the detection threshold, even at the lowest concentration tested of 0.1 µg/mL (Figure 3G). The fluorescence‐based readout exhibited higher sensitivity than the EOT‐based response, achieving an LoD of 0.1 µg/mL – approximately eight times lower than that of EOT – consistently observed in both PBS and artificial serum (AS) (Figure 3H,I). Moreover, fluorescence demonstrated superior accuracy at low concentrations, with a relative standard deviation (RSD) of just 0.8%.

Remarkably, both EOT and fluorescence signals correlate with doxorubicin concentration across overlapping ranges. This agreement provides built‐in cross‐validation, thereby reducing false positives and minimizing signal drift. Fluorescence detection offers superior sensitivity for quantifying low drug concentrations (sub‐µg/mL), while the interferometric method provides a broader dynamic range suitable for higher concentrations (up to tens of µg/mL). Since chemotherapeutic regimens demand accurate monitoring across this entire range, the dual readout ensures reliable quantification under clinically relevant conditions, thereby supporting precision chemotherapy and mitigating the risks of therapeutic failure (underdosing) or systemic toxicity (overdosing).

Time‐lapse imaging under accelerated conditions (PBS, pH 7.4, 45°C) with the sensor sandwiched between artificial skin flaps revealed progressive disintegration culminating in complete dissolution of the device (Figure 3L). This process results from the sequential breakdown of the constituent materials. The porous silica (PSiO_2_) scaffold undergoes hydrolytic dissolution into soluble silicic acid (Si(OH)_4_), which is safely cleared through renal excretion [54]. The PLGA substrate degrades via ester bond hydrolysis into lactic and glycolic acids, both naturally metabolized through the Krebs cycle [16, 55]. The conformal PPy‐based MIP follows the fragmentation pathway of its underlying scaffold, degrading in parallel without interfering with the resorption of PSiO_2_/PLGA [56]. All components are well established as biocompatible in a broad range of in vivo applications [57, 58, 59]. To specifically assess byproduct safety under physiological conditions, the MIP sensor was immersed in PBS (pH 7.4) and 37°C, and aliquots collected over 0, 30, 60, and 90 days were analyzed by HPLC–UV–vis. No detectable pyrrole release was observed at any time point, irrespective of the progressive degradation of the sensor, confirming that PPy degradation proceeds without generating measurable toxic residues under physiologically relevant conditions.

**FIGURE 4:**
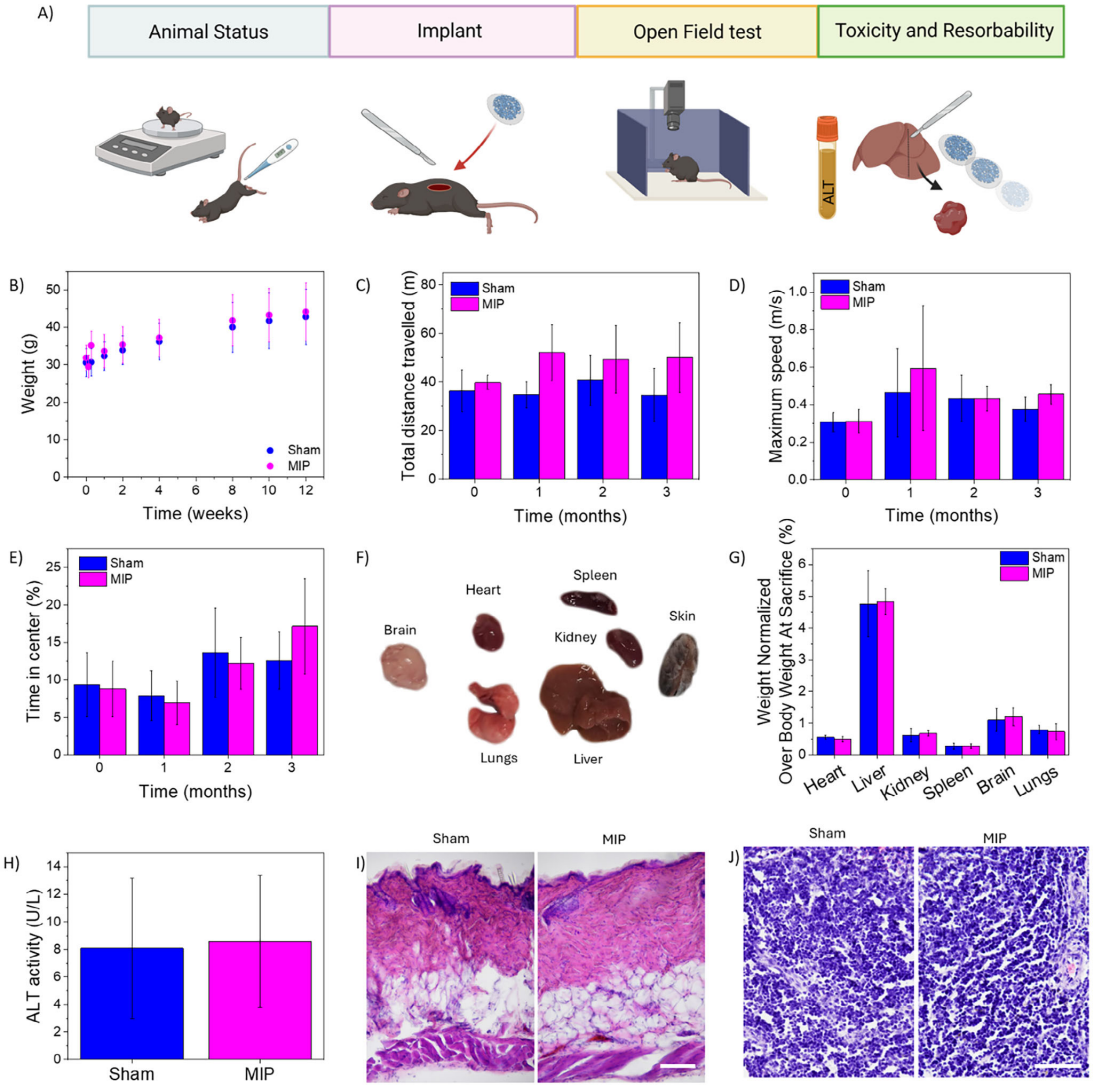
In vivo systemic and local assessment of biocompatibility and bioresorbability of MIP sensor. A) Schematic of the in vivo protocol, including animal status evaluation, subcutaneous biosensor implantation, open‐field behavioral testing, and toxicity/resorbability assessment via serum and organ analysis. B) Weight gain expressed over body weight at baseline (%) of Sham (n = 7) and sensor‐implanted mice (n = 7) from 24 h to the 12th week after implantation. Weight gain during the experimental period is not different between experimental groups. C–E) Total distance travelled (C), maximum speed (D), and % on time spent in the center (E) in the OF test at baseline and 4, 8, and 12 weeks after implantation. F) Images of organs after 3 months from implantation: spleen, heart, kidney, lungs, brain, liver, and skin. G) Organ weight normalized over body weight (%) at sacrifice of Sham (n = 7) and sensor‐implanted mice (n = 7). H) ALT activity (U/L) as an index of hepatic function in Sham (n = 7) and MIP sensor (n = 7) mouse groups. I) Histological images of skin from Sham (left panel) and sensor‐implanted mice (right panel) showing normal histological structures. Scale bar = 100 µm. J) Histological images of spleen from Sham (left panel) and sensor‐implanted mice (right panel) showing normal histological structures. Scale bar = 100 µm. Data in (B–E, G, H) are shown as mean ± SD and analysed according to repeated measures ANOVA, with time and type of implant as factors and sex as covariate.

To evaluate the in vivo safety and biocompatibility of the MIP sensor, we conducted a comprehensive 12‐week study in mice implanted with the sensor and sham control mice. A range of parameters including general integrity, behavior, body weight and temperature, organ function, and histological assessment, were monitored to assess both systemic toxicity and local tissue response (Figure 4A).

Weekly evaluation of animal integrity using the SHIRPA test, such as monitoring activity status, tremor, lacrimation, eyelid closure, fur appearance, whisker movement, and defecation, revealed no alterations of the animal status, indicating the lack of sensor‐dependent systemic toxicity (Table S3). Body weight increased physiologically over time with no statistically significant differences between groups (Figure 4B; Table S4). Body temperature remained stable and comparable across groups at all monitored timepoints (24, 48, 72 h, 7 and 14 days post‐implantation), excluding an acute inflammatory response (Figure S10, Table S4). Systemic toxicity was also investigated performing the open field behavioral test (OF), before surgery (baseline) and 4, 8, and 12 weeks after implantation (Figure 4C–E; Table S4). Total distance travelled and maximum speed (Figure 4C,D), indices of spontaneous locomotor activity, as well as anxiety, indirectly measured as percentage of time spent in the center (Figure 4E), were not affected in implanted animals compared to sham mice, further indicating the absence of sensor‐dependent toxicity.

At the study endpoint, retroorbital blood samples were collected. Subsequently, the mice were sacrificed, and major organs (liver, spleen, kidney, heart, lung, brain, and skin) were collected and examined (Figure 4F; Table S4). Organ weights normalized to body weight showed no significant differences between mice implanted with the MIP sensor and sham mice (Figure 4G), and no alteration in gross morphology was observed. In addition, to assess MIP biodegradation, the implantation site was examined at the time of sacrifice by carefully incising and lifting the skin. Serum levels of alanine aminotransferase (ALT), a marker of hepatic toxicity, were also comparable between groups, indicating no liver impairment (Figure 4H).

The MIP sensor had mostly disappeared three months after implantation, and only a small residual area (∼1 mm) remained where part of the sensor was visible in 4 out of 7 implanted mice, with no observable macroscopic changes in the surrounding tissues. This suggests that the sensor degradation time under physiological conditions slightly exceeds the three‐month duration of the experiments. Transversal skin and spleen sections were cut with a cryostat, respectively, into 40 and 10 µm slices and colored with haematoxylin and eosin staining. Histological structure and cellular density of spleen and skin were considered. Consistently, results confirmed the physiological phenotype of skin and spleen in both sham and sensor‐implanted mice (Figure 4I,J).

## 3 Conclusions

We have developed and validated a fully bioresorbable, dual‐readout optical sensor capable of selective, quantitative detection of the chemotherapeutic agent doxorubicin. By integrating a nanostructured porous silica scaffold with a conformal MIP synthetic receptor, the sensor achieves specific molecular recognition based on shape and chemical complementarity over a clinically relevant operation time for chemotherapeutic treatment of 12 weeks in vitro. The combination of effective optical thickness shifts and changes in the fluorescence intensity provides a robust, internally self‐validating readout across a wide concentration range. Compared to single‐mode systems, this multiparametric architecture significantly enhances both analytical sensitivity and measurement fidelity, particularly in biologically complex environments (Table S1).

The MIP sensor demonstrated excellent reproducibility, reversibility, and operational stability under physiologically relevant conditions, with a limit of detection down to 0.1 µg/mL. In vivo implantation in a murine model confirmed its long‐term biocompatibility and degradation within 12 weeks, without eliciting adverse systemic or local responses.

As summarized in Table S5, earlier MIP‐based sensors are generally limited to single‐mode readout, evaluate doxorubicin over similar or narrower concentration ranges, and do not include in vivo biocompatibility studies or any assessment of biodegradability.

This work represents a significant advance in the field of transient bioelectronics and in vivo chemical sensing. The modular nature of the platform, along with its compatibility with customizable MIP chemistries, makes it readily extensible to a broad class of small‐molecule therapeutics and soluble biomarkers. By enabling localized, real‐time drug monitoring without requiring device retrieval, this sensor lays the groundwork for next‐generation implantable diagnostics tailored to adaptive and personalized cancer treatment strategies.

Looking ahead, clinical translation can be facilitated by pairing the bioresorbable implant with an external wearable electronic patch equipped with near‐infrared (NIR) and fluorescence excitation sources and photodiode detectors [31]. Such an external patch would enable a fully non‐invasive, real‐time readout of the implant's optical signals through the skin, eliminating the need for surgical retrieval and significantly improving patient safety and comfort. The combination of a biodegradable implant and a reusable wearable reader thus provides a tangible technological route toward precision, patient‐tailored chemotherapy monitoring, aligning with the vision of intelligent, transient bioelectronic systems for adaptive oncology.

## 4 Materials and Methods

### 4.1 Materials and Chemicals

The substrate used for the preparation of the bioresorbable optics of this report was a single‐side polished Si boron‐doped wafers (p‐type) with resistivity of 0.8–1.2 mΩ×cm and orientation <100> (Siltronix Silicon Technologies, France). Aqueous hydrofluoric acid (HF, 48%), sodium hydroxide (NaOH, 98%), aqueous hydrochloric acid (HCl, 37%), sodium acetate (CH_3_COONa, 99%), sodium phosphate monobasic monohydrate (PBS, 98%), tris(hydroxymethyl)aminomethane (TRIS, 99%), sodium chloride (NaCl, 99%), chloroform (CHCl_3_, > 99%), poly(D,L‐lactide‐co‐glycolide) (PLGA, 85:15 lactide:glycolide, Mw = 50 000–75 000), potassium chloride (KCl, 99%), sodium bicarbonate (NaHCO_3_, 99,7%), sodium chloride (NaCl, 99%), were purchased from Merk‐Sigma–Aldrich (Germany). Monosodium phosphate (MSP), NaH_2_PO_4_, and disodium phosphate (DSP), Na_2_HPO_4_, were provided from Honeywell Fluka (College Park, GA, USA) Poly(allylamine hydrochloride) (PAH, Mw = 17 000) and poly(methacrylic acid sodium salt) (PMAA, Mw = 18 600) were purchased from Merck (Germany). Absolute ethanol (EtOH, 99.9%), isopropyl alcohol (IPA, 99.5%), and diethyl ether (Et_2_O, > 99 were purchased from Carlo Erba Reagents. Pyrrole (reagent grade, 98%), iron(III) chloride hexahydrate (97%), doxorubicin hydrochloride (98.0%–102.0%), paclitaxel (from semisynthetic, ≥98%), 5‐fluorouracil (≥99%), and cyclophosphamide monohydrate (≥99%) were used as provided from Sigma–Aldrich. Betadine, and Neuflan were purchased from the pharmacy of the hospital of Modena (Italy). Isoflurane (Vetflurane, 1000 mg/g) was purchased from Virbac (Italy). Histo‐mount was purchased from HIsto‐Lab (Italy). Sucrose (99%) was purchased from Thermo Fisher Scientific (USA). Aqueous solutions were prepared in deionized water (DIW, 15 MΩ×cm) filtered by Elix (Merck Millipore). Neuflan gel (Neomycin 0.5 g/Flucinolone acetonide 0.025 g/Lidocaine 2.5 g. hematoxylin and eosin. All buffers were prepared in DIW, and their pH adjusted with NaOH (5 m) and HCl (5.4 m) aqueous solution.

### 4.2 Preparation of Porous Silica (PSiO_2_) Layer on Silicon Substrate

Porous silicon (PSi) layer was prepared at room temperature (RT) by two‐step electrochemical etching in 2 mL of a HF:EtOH (3:1 v/v) aqueous solution of p^++^ (0.8–1.2 mΩ×cm) silicon substrate (1.5 cm by 1.5 cm). A custom‐made Teflon cell equipped with a platinum wire cathode and a flat aluminum anode was used to etch silicon over a circular area of 0.567 cm^2^. A source measurement unit (Keithley 2602A) was employed to set the etching current. The first etching step was conducted at a current density of 300 mA cm^−^^2^ for 25 s, producing a high‐porosity PSi layer. The samples were then rinsed with EtOH for 120 s to remove residual HF, followed by immersion in a NaOH (1 M):EtOH (9:1 v/v) solution for 120 s to create a nanostructured surface and prevent the formation of parasitic layers. Finally, samples were rinsed in deionized water (DIW) and EtOH to remove traces of the dissolving solution. In the second step, the etching time was increased to 40 s, followed by rinsing in EtOH for 120 s and in Et_2_O for 120 s to obtain a crack‐free high‐porosity PSi layer. The etching current density and time were optimized through process calibration, yielding a porosity of 76% ± 1%, an average pore diameter of ∼20 nm, and a PSi membrane thickness of 5.3 ± 0.1 µm in the second etching step (Figures S2 and S3). The porosity value was selected as the maximum value compatible with maintaining mechanical stability of the membrane, thereby ensuring the largest possible pore diameter after thermal oxidation and facilitating efficient molecular diffusion during subsequent functionalization and sensing experiments. In fact, porosity decreased from ∼76% to ∼53% upon oxidation, owing to the volume expansion of the silicon skeleton during conversion to SiO_2_.

The PSi membrane was eventually converted into PSiO_2_ by thermal oxidation in a muffle furnace (ZB/1, ASAL) at 1000°C for 5 min. These conditions increase the stability of the nanostructured PSiO_2_ membrane under physiological conditions by reducing its dissolution rate [27].

### 4.3 Preparation of PLGA Foil

A polymer solution was prepared by dissolving 0.2 g of PLGA pellets in 10 mL of chloroform at room temperature (RT) under gentle stirring for 6 h, with a glass cover placed to minimize solvent evaporation. The resulting solution was cast onto a 9 cm glass Petri dish, which was immediately covered and left overnight in an air‐saturated environment with chloroform vapors to allow slow solvent evaporation. The formed PLGA film (∼50 µm thick) was detached by immersion in deionized water (DIW) and manually peeled from the dish. The film was then dried in a ventilated oven (G‐Therm 035, Fratelli Galli, Italy) at 30°C for 5 min prior to use.

### 4.4 Preparation of the PSiO_2_ Membrane on a PLGA Foil

A two step electrochemical etching was performed on silicon substrates as reported in Section 4.2 (*Preparation of Porous Silica (PSiO_2_) Layer on Silicon Substrate*). A circular contour was then scratched into the PSi surface using a diamond tip to delimit the area of interest. The PSi layer was then detached from the surrounding native Si substrate via an electropolishing step performed in a Teflon cell at a current density of 800 mA cm^−^^2^ for 0.1 s. An etching solution of HF:EtOH (1:1, v/v) with reduced HF concentration was used to facilitate electropolishing. The released PSi membrane was rinsed sequentially in EtOH and Et_2_O and sandwiched between two non‐polished silicon dices. Conversion into PSiO_2_ was achieved by thermal oxidation at 1000°C for 5 min in a muffle furnace (ZB/1, ASAL, Italy). Following oxidation, the upper silicon dice was removed and a PLGA foil (3 × 2 cm, 50 µm thickness) was placed on top of the PSiO_2_ membrane. The PLGA–PSiO_2_–Si assembly was heated at 100°C for 60 s on a hot plate (ARE 230 V, Velp Scientifica, Italy), then rinsed in DIW to release the PLGA–PSiO_2_ film from the supporting Si substrate. Finally, the composite membrane was gently dried at 30°C for 5 min in a ventilated oven (G‐Therm 035, Fratelli Galli, Italy).

### 4.5 Morphological Characterization

Surface morphological characterization of PSiO_2_ and MIP PSiO_2_ sensors was performed using a field emission scanning electron microscope (FEG‐SEM, Zeiss SUPRA) at an acceleration voltage of 10 kV and various magnifications. The pore size distribution was determined by analyzing top‐view SEM images using the Gwyddion software, resulting in an average change of the pore diameter from 21±3 to 17±6 nm, which results in a MIP thickness of about 2 nm.

### 4.6 Optical Characterization of PSi and PSiO_2_ Layers

Reflectance spectra of PSi and PSiO_2_ layers were acquired in the spectral range 400 – 1000 nm in air. Light exiting from a deuterium‐tungsten halogen lamp (HL‐2000, Ocean Optics, USA) was shed through one arm of a bifurcated fibre‐optic probe (QR200−7‐VIS‐BX, Ocean Optics, USA) onto the sample surface. The reflected light was collected through the second arm of the fibre probe and guided into a UV–vis spectrometer (SM242, Spectral Products, USA). Reflectance spectra were collected with an integration time of 50 ms, an acquisition interval of 10 ms and a spectral resolution of 0.35 nm. The line‐shape of the light source spectrum was corrected through normalization with respect to the reflectance spectrum of a silver mirror (PF10‐03P01‐Ø 25.4 mm, Thorlabs, USA). Undesired contributes due to the environmental light were also subtracted.

Porosity, thickness, and effective optical thickness (EOT) of the PSi and PSiO_2_ layers were measured by best fitting a Fabry–Pérot interferometer model to the reflectance data using home‐made software routines [34].

### 4.7 Functionalization of the PSiO_2_ Surface with Molecularly Imprinted Polymer (MIP) Films

First, 50 µL of poly(allylamine hydrochloride) (PAH, 1 mg mL^−^^1^ in TRIS buffer, pH 8) was dispensed onto the PSiO_2_ surface and incubated for 1 h, forming a positively charged PAH layer electrostatically bound to the negatively charged SiO_2_ surface. Subsequently, a negatively charged layer of poly(methacrylic acid sodium salt) (PMAA, 1 mg mL^−^^1^ in TRIS buffer, pH 8) was deposited by drop‐casting 50 µL of solution and incubating for 1 h, enabling electrostatic binding to the underlying PAH layer. These conditions optimized the deposition of nanometer‐thick polyelectrolyte layers, facilitating subsequent functionalization steps while preserving the pore diameter [27]. Next, 50 µL of a doxorubicin solution (0.15 mg mL^−^^1^ in PBS, pH 5.8) was drop‐cast onto the silica layer and incubated for 1 h, enabling the electrostatic anchoring of positively charged doxorubicin (pK_a1_ = 8.1) molecules to the negatively charged PMAA layer. A volume of 5 µL of a FeCl_3_ in ethanol (1.5% w/v) was then drop‐cast onto the modified PSiO_2_ layer and allowed to evaporate for 5 min. This procedure was repeated four additional times to ensure a uniform distribution within the PSiO_2_ scaffold of ferric chloride, which acts as the oxidizing agent for the subsequent vapor‐phase polymerization step [34]. The PSiO_2_ scaffolds were then exposed to saturated pyrrole vapor by placing them atop a 4 mL vial containing 20 µL of pure pyrrole, allowing vapor‐phase polymerization to proceed for 1 h. These conditions optimized the formation of a uniform, nanometer‐thin PPy film conformally coating the inner surface of the PSiO_2_ scaffolds [34]. Finally, the samples were thoroughly rinsed with deionized water for 5 min to remove unreacted monomer and byproducts. Afterward, the substrates were immersed in TRIS buffer (30 mm + 30 mm NaCl) at pH 8.7 for 30 min to remove doxorubicin from the polymer matrix, thereby generating the MIP biosensor. After each functionalization step, reflectance spectra were acquired and the corresponding EOT values were calculated, following the protocol described in Section 4.6 (*Optical Characterization of PSi and PSiO_2_ Layers*), to verify the progression and reproducibility of the functionalization process.

As a control, non‐imprinted polymers (NIPs) on PSiO_2_ scaffolds were synthesized following the same procedure used for MIP sensors, except that polypyrrole (PPy) polymerization was performed without prior binding of the target molecule. NIP samples were subjected to the same washing steps as the MIPs to ensure comparable processing conditions.

### 4.8 Preparation of Artificial Serum (AS)

Artificial serum solutions consisted of mixtures of PBS (10 mm), NaCl (110 mm), KCl (3.48 mm), CaCl_2_ (1.53 mm), MgSO_4_ (0.69 mm), NaHCO_3_ (26.2 mm), NaH_2_PO_4_ (1.67 mm), Na gluconate (9.64 mm), glucose (5.55 mm), and sucrose (7.6 mm). The pH solution was adjusted with the pH meter to obtain the pH 7.4.

### 4.9 In Vitro Incubation with Doxorubicin Solutions

MIP sensors and NIP controls were incubated for 1 h with 50 µL of either PBS (10 mm + 100 mm NaCl at pH 7.4) or AS solutions with doxorubicin at concentrations in range from 0.1 to 150 µg/mL. After incubation at a given doxorubicin concentration, the samples were rinsed in DIW for 30 s and dried under a gentle nitrogen flux.

### 4.10 Characterization of the Sensor Using Reflectance Spectroscopy

The MIP sensor and the NIP control on the native silicon substrate were characterized by recording reflectance spectra and calculating effective optical thickness (EOT) values in the near‐infrared range (600–950 nm), both before and after exposure to doxorubicin solutions at defined concentrations ranging from 0.1 to 150 µg/mL.

The effect of incubation time on doxorubicin binding to the MIP sensor was assessed over a range of 5–120 min using a fixed doxorubicin concentration of 5 µg/mL in phosphate‐buffered saline (PBS) at pH 7.4. Freshly prepared standard solutions (50 µL) were drop‐cast onto the sensor surface and incubated for the designated durations. After incubation, the sensors were washed with deionized water for 3 min under stirring, rinsed with ethanol, and dried under a nitrogen stream. Prior to each measurement, the biosensors were regenerated by immersion in TRIS buffer pH 8.7 for 10 min under stirring.

For sensing experiments, the MIP sensor and NIP control were exposed to increasing concentrations of doxorubicin standard solutions prepared in phosphate‐buffered saline (PBS) or artificial serum (AS). Incubations were performed in a closed chamber to minimize evaporation.

For all the experiments, reflectance spectra were recorded before and after each concentration step, from which EOT values were calculated. All experiments were conducted in triplicate (n  =  3).

Binding performance was evaluated by fitting the dose–response curves (ΔEOT vs. doxorubicin concentration) to a Langmuir adsorption model:

$$\Delta EOT\left(x\right)=\frac{ax}{b+x}$$

where ΔEOT is the optical response, *x* is the doxorubicin concentration (µg/mL), a represents the theoretical maximum response (saturation plateau), and *b* is the concentration at which half‐maximal binding occurs. The apparent association constant (K_0_) was calculated from the fitted parameter *b* using:

$$K_{0}=\frac{1}{b}$$

The sensitivity of MIP sensor and NIP control was estimated from the slope of their respective calibration curves in the linear region. The limit of detection (LoD) was calculated based on the standard deviation (SD) of the background noise and the sensitivity according to the following formula:

$$LoD=\frac{3\sigma }{S}$$

where σ is the SD of the EOT signal acquired before doxorubicin incubation, and *S* is the sensitivity.

### 4.11 Characterization of the Sensor Using Photoluminescence Spectroscopy

Photoluminescence spectra of MIP sensor on PLGA foil were collected in the wavelength range 500 – 700 nm in air, using a tunable laser at 470 nm with 20 nm of band. The beam impinged the bottom surface of the PLGA foil with an angle of ∼30° while the emitted light was collected through a lens‐terminated optical fiber (CVH100‐COL, Thorlabs, USA), this latter connected to a UV–vis spectrometer (USB2000+ UV–vis, Ocean Optics, USA). The photoluminescence spectra were recorded with an integration time of 1 s and a spectral resolution of 0.35 nm.

The calibration curve was obtained by plotting the PL peak intensity at 590 nm vs the doxorubicin concentration. The sensitivity (*S*) was defined as the slope of the linear regression line fitted to the experimental data. The limit of detection (LoD) was calculated based on the standard deviation (SD) of the background noise and the sensitivity according to the following formula:

$$LoD=\frac{3\sigma }{S}$$

where σ is the SD of the PL signal acquired before doxorubicin incubation, and *S* is the sensitivity.

### 4.12 Selectivity, Repeatability, and Stability Tests

MIP selectivity was evaluated by exposing the sensor to solutions containing structurally related chemotherapeutic agents—paclitaxel (PA), cyclophosphamide (CY), and fluorouracil (5‐FU)—at concentrations ranging from 0.1 to 5 µg/mL.

Repeatability was assessed by performing three consecutive doxorubicin detection experiments using the same MIP sensor. Between each run, the sensor was regenerated by treatment with TRIS buffer at pH 8.7 for 10 min under stirring.

MIP sensor stability over time was investigated by monitoring the response to 2 µg/mL doxorubicin at multiple time points over a 90‐day period. Prior to each measurement, the sensor was reconditioned using the same TRIS buffer at pH 8.7 regeneration protocol.

### 4.13 Accelerated Degradation Tests in Vitro

To assess the degradation profile of the MIP‐based sensor in a physiologically relevant environment, MIP sensors were positioned between two flaps of artificial skin (SynDaver, USA) and incubated at 45°C in phosphate‐buffered saline (PBS, pH 7.4) to accelerate hydrolytic degradation. Images were captured at predefined time points using a digital camera under standardized lighting conditions. The degradation process was qualitatively evaluated by monitoring changes in the physical integrity and visibility of the sensor through the artificial skin over time.

### 4.14 HPLC Analysis of Pyrrole Release During Sensor Degradation

To evaluate the possible release of pyrrole (Py) monomer during degradation of the MIP–PSiO_2_/PLGA sensors, we incubated the sensors in phosphate‐buffered saline (PBS, pH 7.4) at 37°C under static conditions for 90 days and incubation solution aliquots (100 µL) were collected at defined time points (0, 30, 60, and 90 days) and analyzed by HPLC/UV–vis. HPLC measurements were performed on a HPLC Agilent 1100 series system (Agilent Technologies, Palo Alto, CA, USA) equipped with a vacuum degasser (G1379A), a binary pump (G1312A), an autosampler (G1313A), and a G1315A diode array detector, which was used in the range 200–500 nm. Five microliters of pyrrole standards or incubation solution were injected into the HPLC system, after filtration through 0.22 µm PVDF membranes, using a C18 reversed‐phase column (250 × 4.6 mm, 5 µm). The mobile phase consisted of acetonitrile:water (40:60, v/v) containing 0.1% trifluoroacetic acid, delivered at a flow rate of 1.0 mL min^−^^1^. Detection was carried out at 230 nm, corresponding to the absorption maximum of pyrrole. Chromatographic data were collected and integrated using Agilent Chemstation software. Peak areas versus pyrrole standard concentrations were employed to construct the calibration curve over the range 0.075–1.5 µm (R^2^ = 0.9975), which is 500–10000 times below the no‐observed‐adverse‐effect level (NOAEL) [60], referred to animal used in in vivo experiments in this work (Section 4.16 *In Vivo and Ex Vivo Experiments*).

### 4.15 Computational Predictions of the Pre‐Polymerization Complex

The 2D structures of doxorubicin and pyrrole (Py) were retrieved from the PubChem database (https://pubchem.ncbi.nlm.nih.gov) and converted into 3D conformations using Chem3D software. Subsequent geometry optimization was performed using density functional theory (DFT) calculations implemented in Gaussian16. The optimization employed the B3LYP functional in combination with the 6–311++G(d,p) basis set, ensuring convergence to the lowest‐energy geometries.

Following geometry optimization, molecular mechanics (MM) calculations were conducted using the AMBER force field to quantify the interactions between the monomer and the template. These calculations accounted for the primary non‐covalent forces—hydrophobic interactions, hydrogen bonding, and van der Waals forces—that govern molecular recognition. The cumulative contribution of these interactions was expressed as the overall binding energy (Δ𝐸_binding_), calculated according to the following equation:

$$\Delta E_{\mathrm{binding}}=E_{\mathrm{complex}}\;-\left(E_{\mathrm{template}}+E_{\mathrm{monomer}}\right)$$

where *E*_complex_ is the total energy of the monomer‐template complex*, E*_template_ and *E*_monomer_ are the energies of the individual components in their optimized conformations.

MM simulations between doxorubicin and the pyrrole (Py) monomer were performed using AutoDock Vina to evaluate their intermolecular interactions. The docking results provided insights into the binding affinity of the template–monomer complex prior to polymerization, as well as the types and positions of non‐covalent interactions formed. These outcomes were further analyzed using AutoDockTools 1.5, which enabled detailed examination of bonding characteristics. Visualizations and additional structural analysis of the docking conformations were conducted using UCSF Chimera.

### 4.16 In Vivo and Ex Vivo Experiments

#### 4.16.1 Animals

Three‐month‐old, at study onset, CD1 (n = 14) male and female mice were used. Experimental animals were purchased from Charles River Laboratories Italia s.r.l. (Calco‐Milano, Italy) and housed in individually ventilated transparent cages in a pathogen free facility. Mice were kept in conditioned rooms with stable temperature (21°C±0.5°C) and humidity (60%), on a 12 h light/dark cycle with food and water available ad libitum. All animal procedures were approved by the Committee on Animal Health and Care of the University of Modena and Reggio Emilia and conducted in accordance with National Institutes of Health guidelines [CEE Council 89 609; Italian DL 26/2014, authorization n° 979/2020/PR]. All efforts were made to minimize animal suffering and to reduce the number of animals used in this study following the principle of 3R's (*Replacement, Refinement, and Reduction*) [61, 62].

### 4.17 Molecular Imprinted Polymer (MIP) Implantation Procedure

Mice were divided into two experimental groups as follows: Sham group (subjected to surgery but not to MIP sensor implantation, n = 7) and MIP sensor group (mice implanted with MIP sensors, n = 7). Under general anaesthesia with inhaled isoflurane, animals underwent a surgical operation. Briefly, the upper back skin was disinfected with betadine, lifted, incised, and a subcutaneous pouch of 1.5 × 1.5 cm was created to insert the MIP. The incision was sutured and treated with Neuflan gel (Neomycin 0.5 g/Flucinolone acetonide 0.025 g/Lidocaine 2.5 g) to minimize local post‐operative pain and the risk of infections.

### 4.18 In Vivo Toxicity Evaluation

To evaluate in vivo toxicity, all mice were subjected to a 3 month‐long observational period after surgical procedures according to UNIENISO10993‐6 guidelines. Specifically, different parameters of general integrity were evaluated as follows:

1. Body weight: once a month throughout all the experimental period (3 months); the weight of each animal was measured every 2 weeks and normalized to the weight the animal had prior to surgery.
2. Body temperature: to reveal the occurrence of any inflammatory reaction, rectal temperature was measured before surgery and 6, 24, 48 h (h) and 1 and 2 weeks (w) after surgery.
3. Animal status: a modified SHIRPA test [63] was conducted every two weeks evaluating parameters such as activity status, tremor, lacrimation, eyelid closure, fur appearance, whisker movement and defecation, scored as 0 = absent and 1 = present [7].

Furthermore, to assess locomotion and anxiety, the first behavioral manifestations changing in case of systemic toxicity, the open field (OF) test was performed once a month for 3 months [64, 65]. Briefly, after a 30 min‐long acclimatization in the experimental room, mice were singularly placed in the center of an open wooden chamber (50 × 50 × 40 cm) with dark walls and allowed to explore freely for 10 min. The open space was virtually subdivided into 3 zones, namely, corners (10×10 cm), periphery (within 10 cm of the walls), and center (the rest of the arena). The time spent in the center of the arena was considered as an indirect anxiety index [5, 6]. Travelled distance, maximum speed, and time spent in each zone were automatically recorded and analyzed with ANY‐maze Video Tracking system (Stoelting). The apparatus was thoroughly wiped with 70% ethanol after each test to avoid olfactory cues.

All behavioral observations and tests were performed by an operator unaware of the experimental group to avoid bias.

### 4.19 Ex Vivo Toxicity Evaluation

#### 4.19.1 Sacrifice, Biodegradability Evaluation, Blood and Organ Collection

After 3 months from surgery, mice were sacrificed under general anaesthesia with inhaled isoflurane. Mouse skin surrounding surgery site was cut and lifted to verify, by the necked eye, the presence of some remaining material to assess MIP biodegradability.

Blood was drawn and allowed to clot at room temperature for 30 min and then centrifuged at 1200 g for 15 min to isolate serum. Samples were kept at −20°C until use.

Organs (liver, spleen, kidney, heart, lung, brain, and back skin) were collected and weighed to evaluate any macroscopic alteration. Organs were post‐fixed in 4% paraformaldehyde (PFA) for 48 h and processed for histological analyses.

#### 4.19.2 Histological Analysis of Skin and Spleen

Post‐fixed skin and spleen were immersed in 20% sucrose‐PBS for 2 days, 30% sucrose‐PBS for 3 days and then frozen included in histo‐mount mounting medium using dry ice and stored at −80°C until use. Tissues were sectioned using a cryostat (Leica CM1520) into 40 (skin) and 10 (spleen) µm thick transversal slices and stored on slides at −20°C until use. Slides were air‐dried at room temperature for 4 h prior to hematoxylin and eosin histological staining that was conducted according to validated protocols [27, 31]. Histological images were captured using an optical microscope (Nikon Eclipse Ci with Nikon DS‐Fi3 camera) with a 10X objective.

#### 4.19.3 Hepatic Toxicity Evaluation

As an indicator of hepatic toxicity, serum Alanine aminotransferase (ALT) activity (U/l) was evaluated, according to the manufacturer's instructions (MAK052, Sigma‐ Aldrich). Briefly, ALT activity was assessed through an enzymatic colorimetric assay, using 20 µL of serum per sample, with absorbance reading at 570 nm (Multiskan FC, Thermo Fisher Scientific) and calculated using a linear regression method, with a pyruvate standard curve.

### 4.20 Statistical Analysis

In vitro data are presented as means ± SD values. As concerns in vivo study, data are shown as mean ± SD and analyzed with univariate Analysis of Variance (ANOVA) or two‐way repeated measures ANOVA, as appropriate, using SPSS software (version 26), considering sex as a covariate. Significance threshold was set at *p*‐value (*p*) ≤ 0.05.

## Conflicts of Interest

The authors declare no conflicts of interest.

## Supporting information




**Supporting File**: advs74500‐sup‐0001‐SuppMat.docx.

## Data Availability

The data that support the findings of this study are available from the corresponding author upon reasonable request.
