# Supplementary material for: Multiparametric Bioresorbable Sensor for Doxorubicin Detection via Molecularly Imprinted Synthetic Receptors
Source: Adv Sci (Weinh). 2026 Mar 3;13(30):e24369. doi: 10.1002/advs.202524369 (PMC13248826; doi:10.1002/advs.202524369)
Supplement: Supplementary file 1 — Supporting File: advs74500‐sup‐0001‐SuppMat.docx. [file ADVS-13-e24369-s001.docx]

**Multiparametric Bioresorbable Sensor for Doxorubicin Detection via Molecularly Imprinted Synthetic Receptors**

Martina Corsi^1+^, Tiziano Di Giulio^2+^, Eleonora Vandini^3^, Muhammad Ibrar Asif^2^, Eleonora Daini^3^, Antonietta Vilella^3^, Giuseppina Leo^3^, Alessandra Ottani^3^, Cosimino Malitesta^2^, Daniela Giuliani^3^, Elisabetta Mazzotta^2^*, Giuseppe Barillaro^1^*

^1^Department of Information Engineering, University of Pisa, via G. Caruso 16, 56122 Pisa, Italy

^2^Laboratory of Analytical Chemistry, Department of Biological and Environmental Sciences and Technologies (Di.S.Te.B.A.), University of Salento, via Monteroni, 73100 Lecce, Italy

^3^Department of Biomedical Metabolic and neural Sciences, University of Modena and Reggio Emilia, via G. Campi 287, 41125 Modena, Italy

^+^These authors contributed equally

^*^Corresponding authors: [giuseppe.barillaro@unipi.it](mailto:giuseppe.barillaro@unipi.it), [elisabetta.mazzotta@unisalento.it](mailto:elisabetta.mazzotta@unisalento.it)

The Supporting Information provides additional data supporting the findings reported in this work.


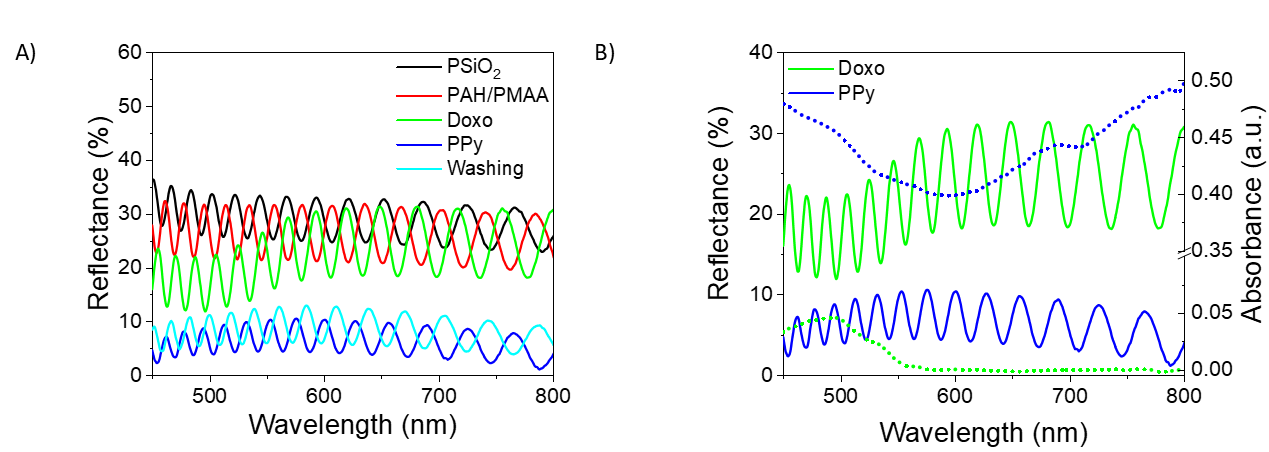


**Figure S1.** A) Reflectance spectra recorded in air on a PSiO_2_ scaffold before and after all the functionalization steps carried out for the synthesis of a PPy-based MIP for doxorubicin. B) Comparison between reflectance spectra recorded in air on a PSiO_2_ scaffold after doxorubicin binding and PPy deposition and absorbance spectrum of doxorubicin solution (green line) and absorbance spectum of PPy (blue line) solution.


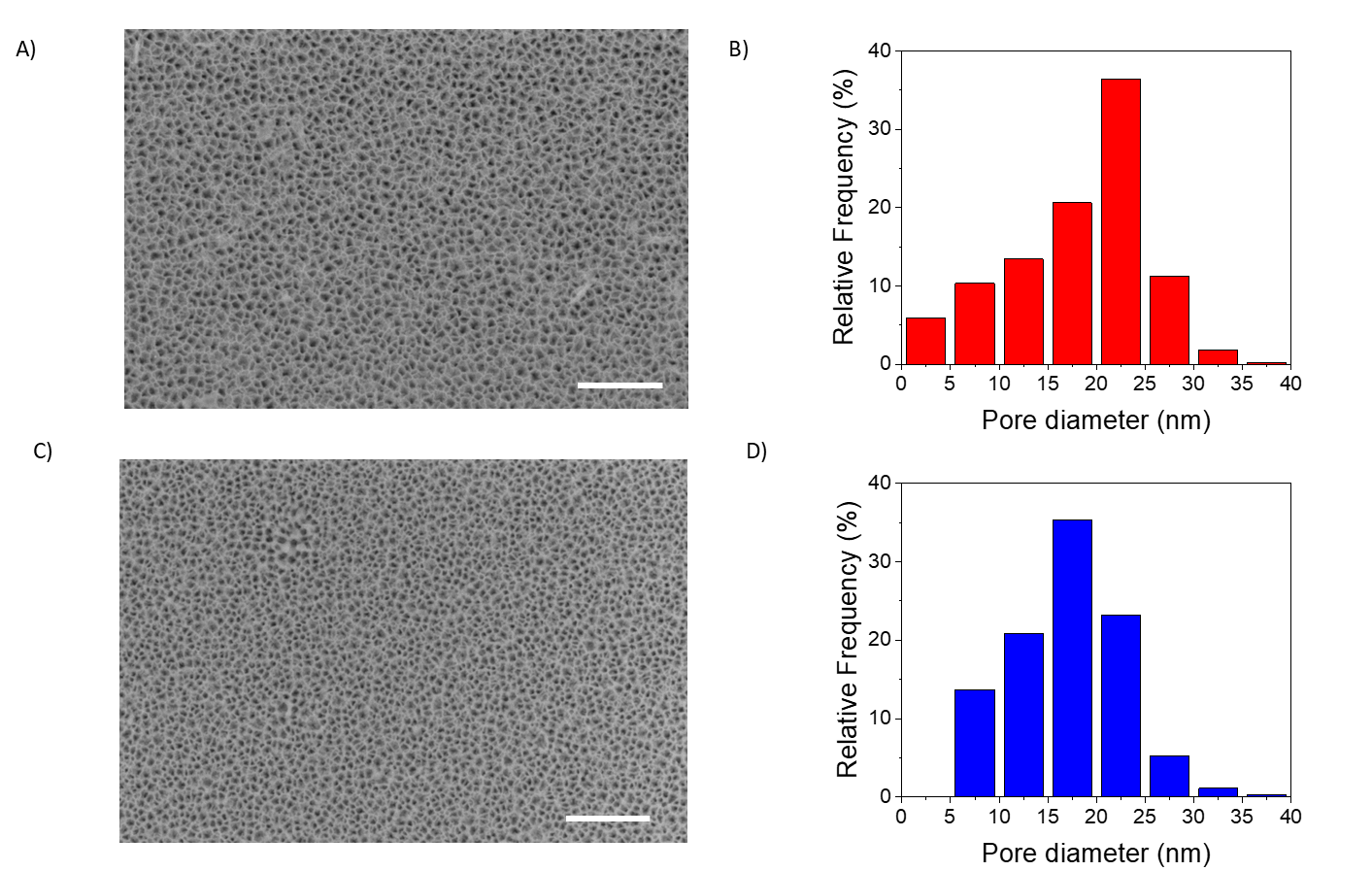


**Figure S2.** A) Top-view SEM image (magnification 120000×) of a PSiO_2_ layer with a thickness of 5 μm and porosity of 76%. Scale bar is 500 nm. B) Distribution of the pore diameter of the PSiO_2_ layer in (A). C) Top-view SEM image (magnification 150000×) of a MIP-PSiO_2_ layer. Scale bare is 500 nm. D) Distribution of the pore diameter of the MIP-PSiO_2_ layer in (C).


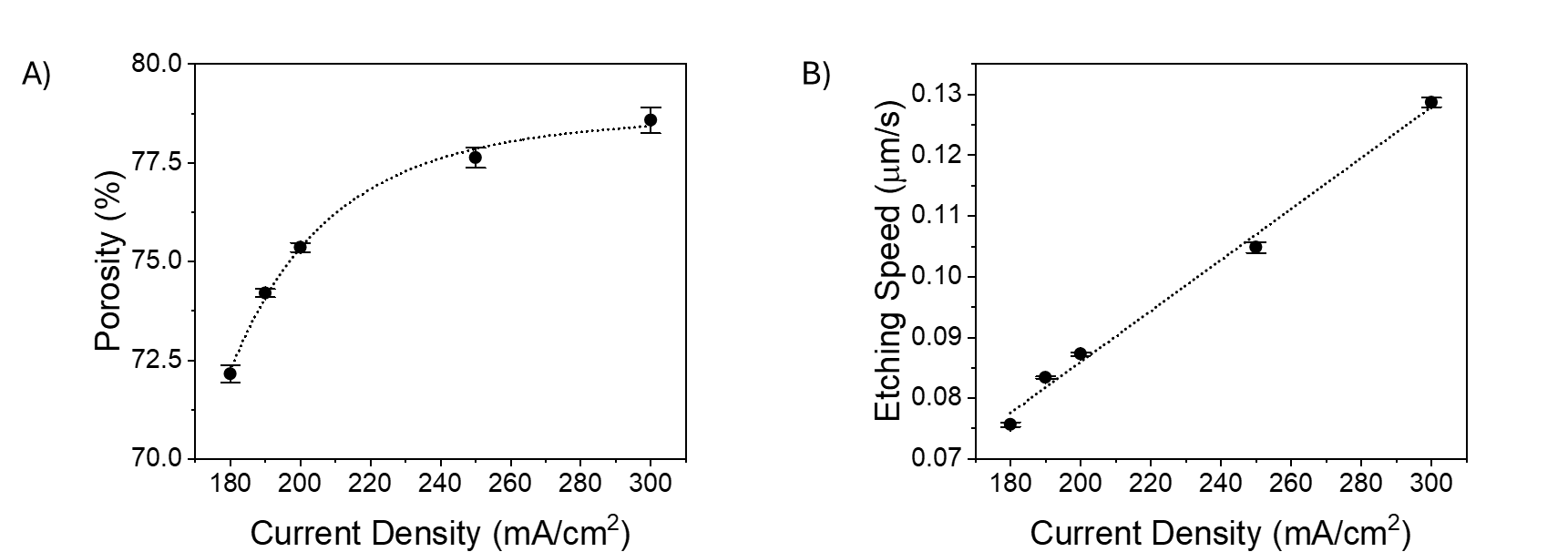


**Figure S3.** Calibration of A) porosity and B) etching rate as a function of current density (180–300 mA/cm²) during porous silicon fabrication. Experimental data are shown as symbols, with solid lines representing the corresponding fits. Each data point represents the average of 4 measurements at a given current density, and error bars indicate the standard deviation.


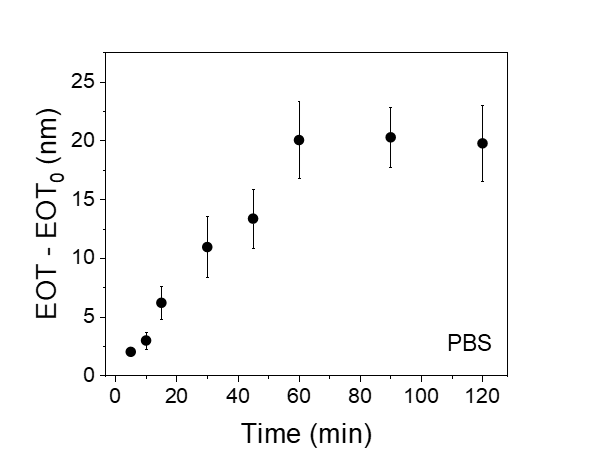


**Figure S4.** EOT-EOT_0_ values related to MIP sensor response time after incubation at 5 μg/mL of doxorubicin in PBS buffer at pH 7.4. The EOT value before incubation (i.e., EOT_0_) is used as reference. Data are reported as the average value measured over 3 devices per each fluid, with error bars representing the standard deviation.


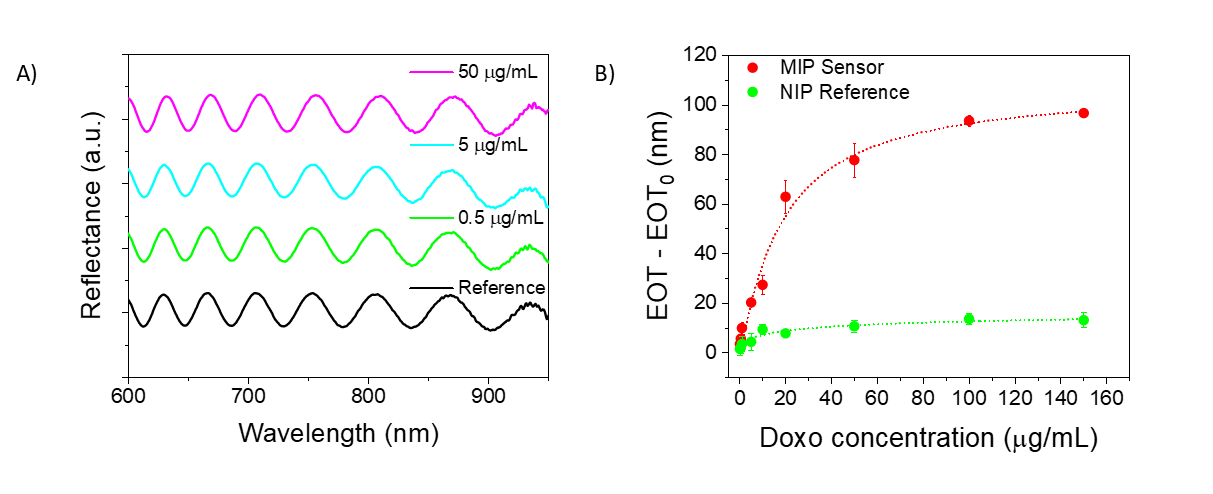


**Figure S5.** A) Reflectance spectra before (reference) and after incubation of doxorubicin at concentration for 0.5,5,50 μg/mL. B) Calibration curves (EOT-EOT_0_ versus doxorubicin concentration) recorded on MIP and not imprinted polymer (NIP) sensors in the range 0.1 to 150 μg/mL. EOT_0_ is measured in buffer solution and AS, used as reference (n_MIP_ = 3, n_NIP_ = 3).


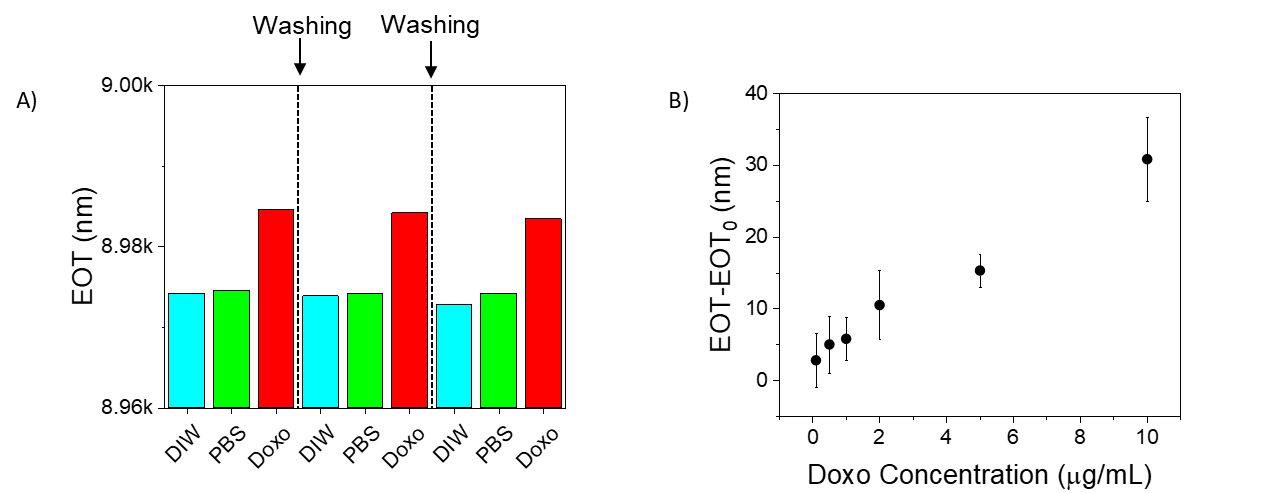


**Figure S6.** A) Reusability test of MIP sensor after incubation with doxorubicin at 2 μg/mL calculated as EOT after washing procedure using TRIS buffer at pH 8.7. B) Reproducibility inter samples represented as EOT-EOT_0_ values versus different doxorubicin concentrations (0.1 μg/mL to 10 μg/mL in PBS buffer at pH 7.4). Data are reported as the average value measured over 3 devices per each fluid with 3 rebinding tests, with error bars representing the standard deviation (n=3).


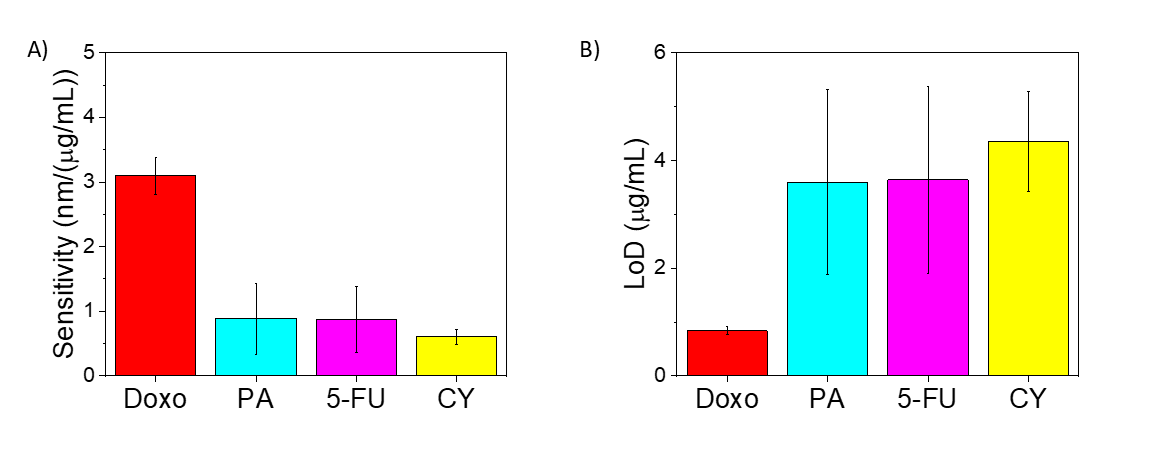


**Figure S7.** A) Sensitivity of the MIP sensor to doxorubicin and to interfering molecules (PA,5-FU, CY). B) LoD of the MIP sensor to doxorubicin and to interfering molecules (PA,5-FU, CY). Data are reported as the average value measured over 3 devices per each fluid, with error bars representing the standard deviation.


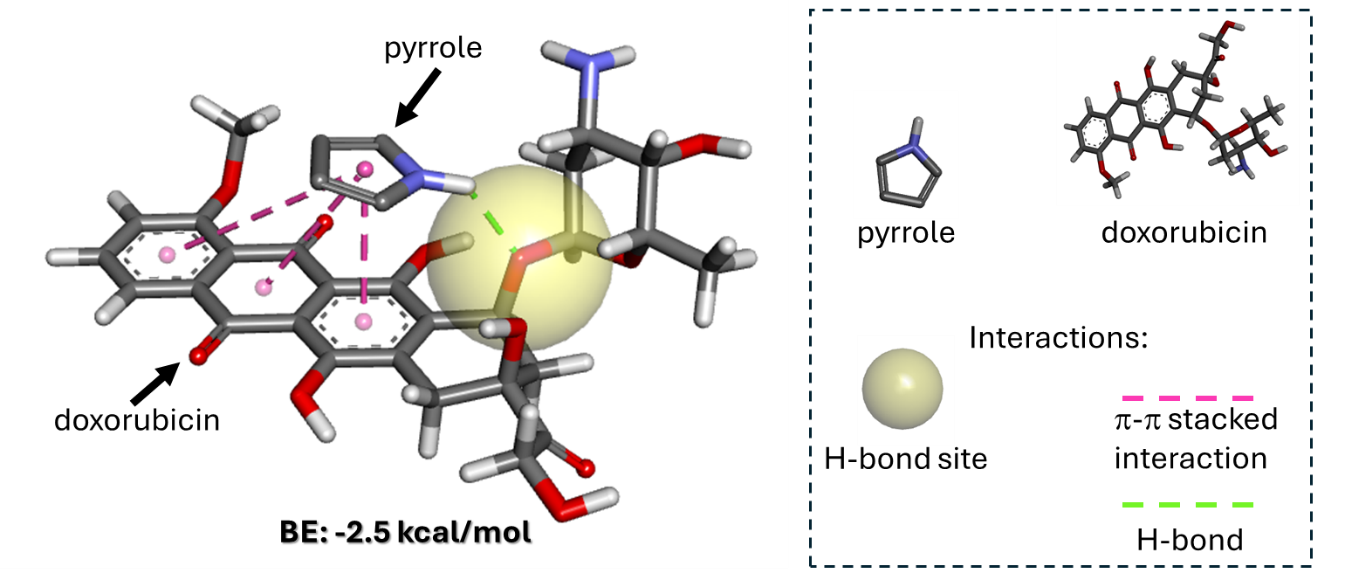


**Figure S8.** Type of interactions and estimated binding energy (kcal/mol) for the pre-polymerization complex between pyrrole monomer and the target doxorubicin. Green dotted lines represent hydrogen bonding, dark pink represents π–π bonds interactions. The H-bond site is indicated by a grey sphere.


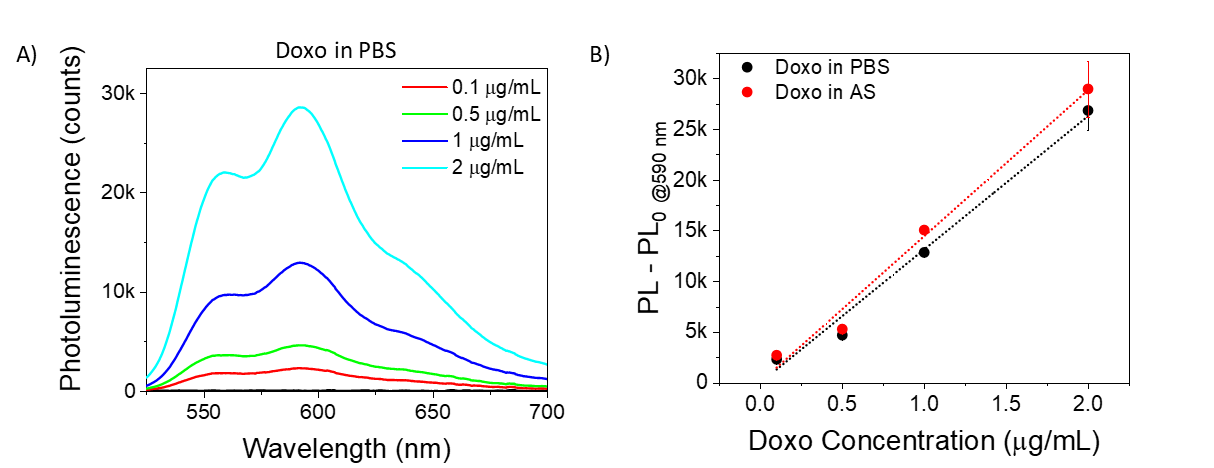


**Figure S9.** A) Sequence of photoluminescence (PL) spectra of PBS solutions of doxorubicin at different concentrations measured in a standard quartz cuvette. B) Calibration curve, namely, PL intensity peak at 590 nm (mean and standard deviation) vs. doxorubicin concentration in PBS and ISF solutions. Dashed lines represent the linear fit to the calibration curve data. PL0 is the intensity value measured for PBS and ISF solutions without doxorubicin. Data in B) are reported as the average value measured over 3 solutions with error bars representing the standard deviation.

**Figure S10.** Rectal temperature of MIP-implanted mice (n=7) and Sham mice (n=7) at 24, 48, 72 h and 7 and 14 days after implantation. Data is shown as mean ± standard deviation and analysed according to repeated measures ANOVA, with time and type of implant as factors and sex as covariate.


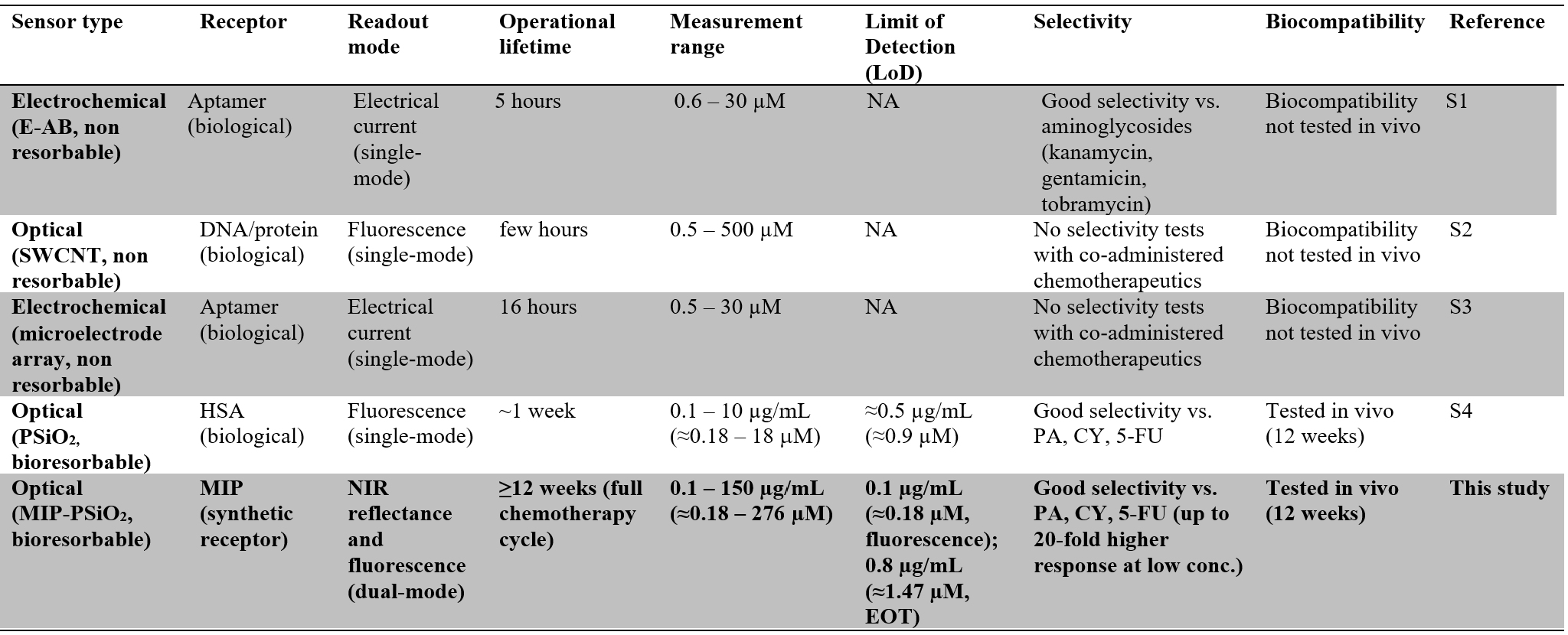


**Table S1.** Comparison of implantable sensors for doxorubicin. E-AB = Electrochemical Aptamer-Based sensor; SWCNT = Single-Walled Carbon Nanotubes; PSiO_2_ = Porous Silicon Dioxide; MIP = Molecularly Imprinted Polymer; HSA = Human Serum Albumin; NIR = Near-Infrared; EOT = Effective Optical Thickness; PA = Paclitaxel; CY = Cyclophosphamide; 5-FU = 5-Fluorouracil; NA = Not Available.


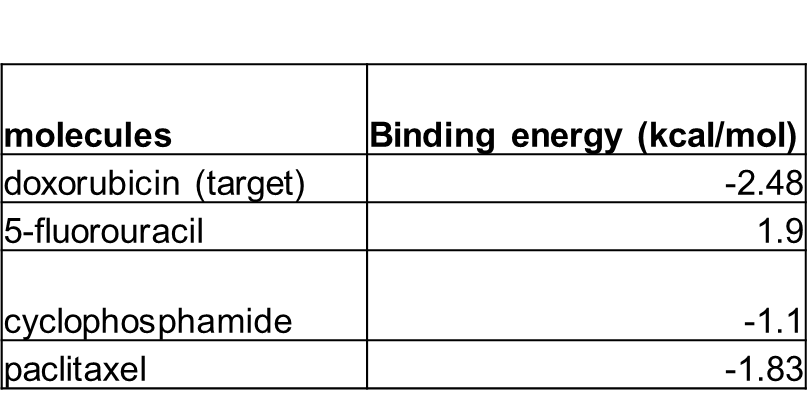


**Table S2.** MM‑calculated binding energies (kcal/mol) for the doxorubicin–pyrrole complex, with analogous energies for pyrrole interactions with other drugs included as controls. Higher negative binding energy values (kcal/mol) indicate better binding affinity.


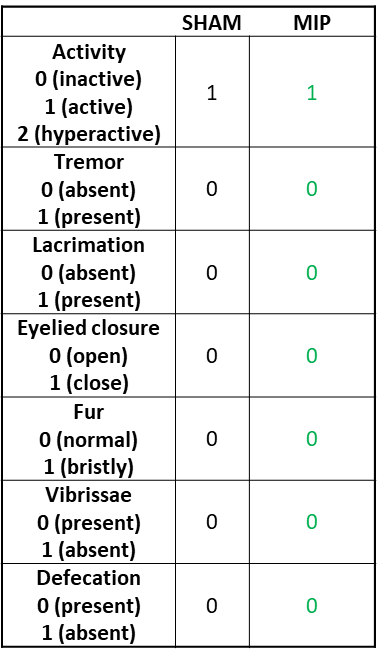


**Table S3.** Animal integrity score using SHIRPA test between sham mice (n=7) and MIP-implanted mice (n=7).


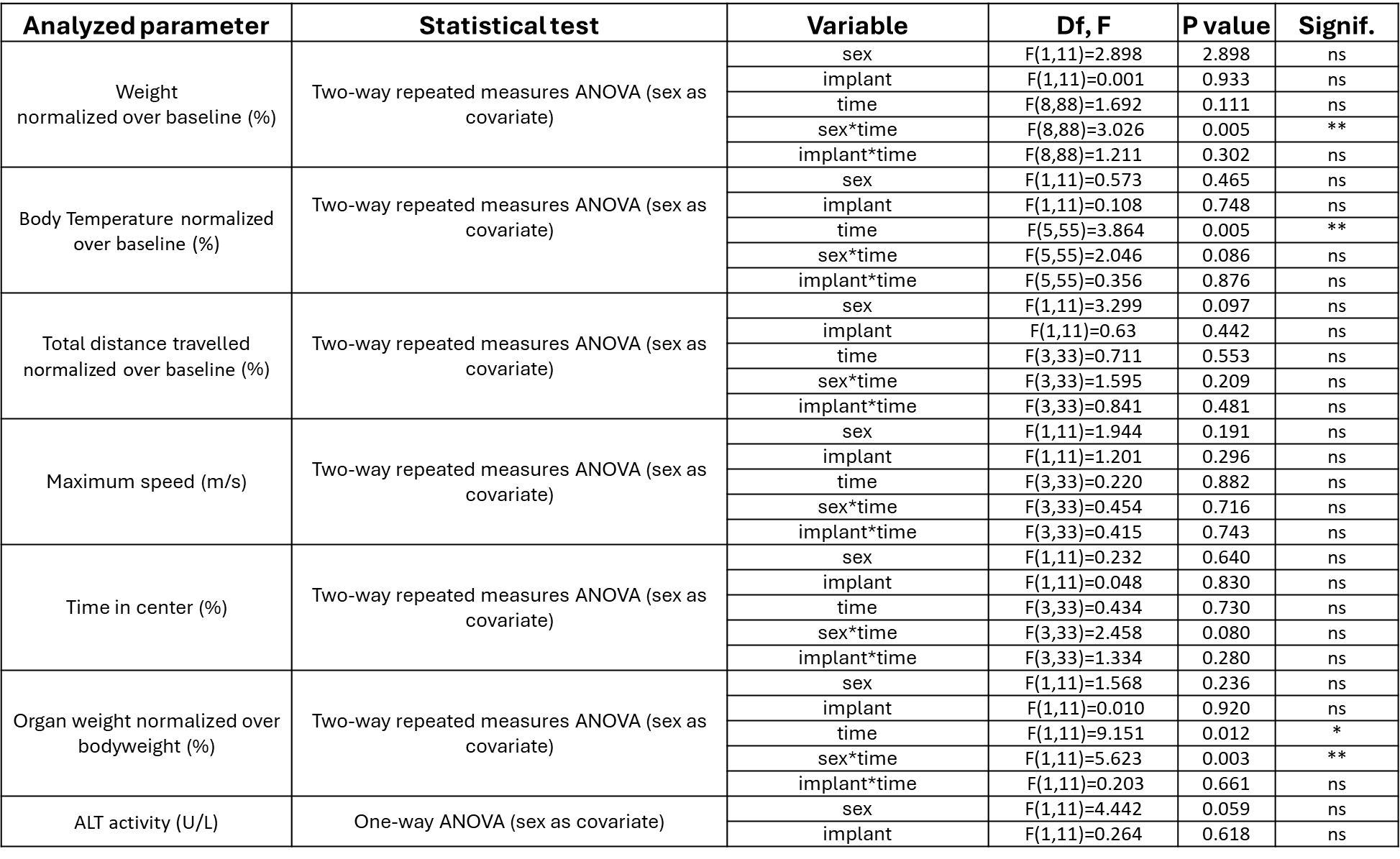


**Table S4.** Statistical analysis of mice during the 3-month observation period and after sacrifice. Statistical tests were performed either on raw data or on data normalized to the baseline, as indicated within the table. Significance levels are expressed as follows: * p ≤ 0.05, ** p ≤ 0.01, *** p ≤ 0.001, ns = not significant.

**
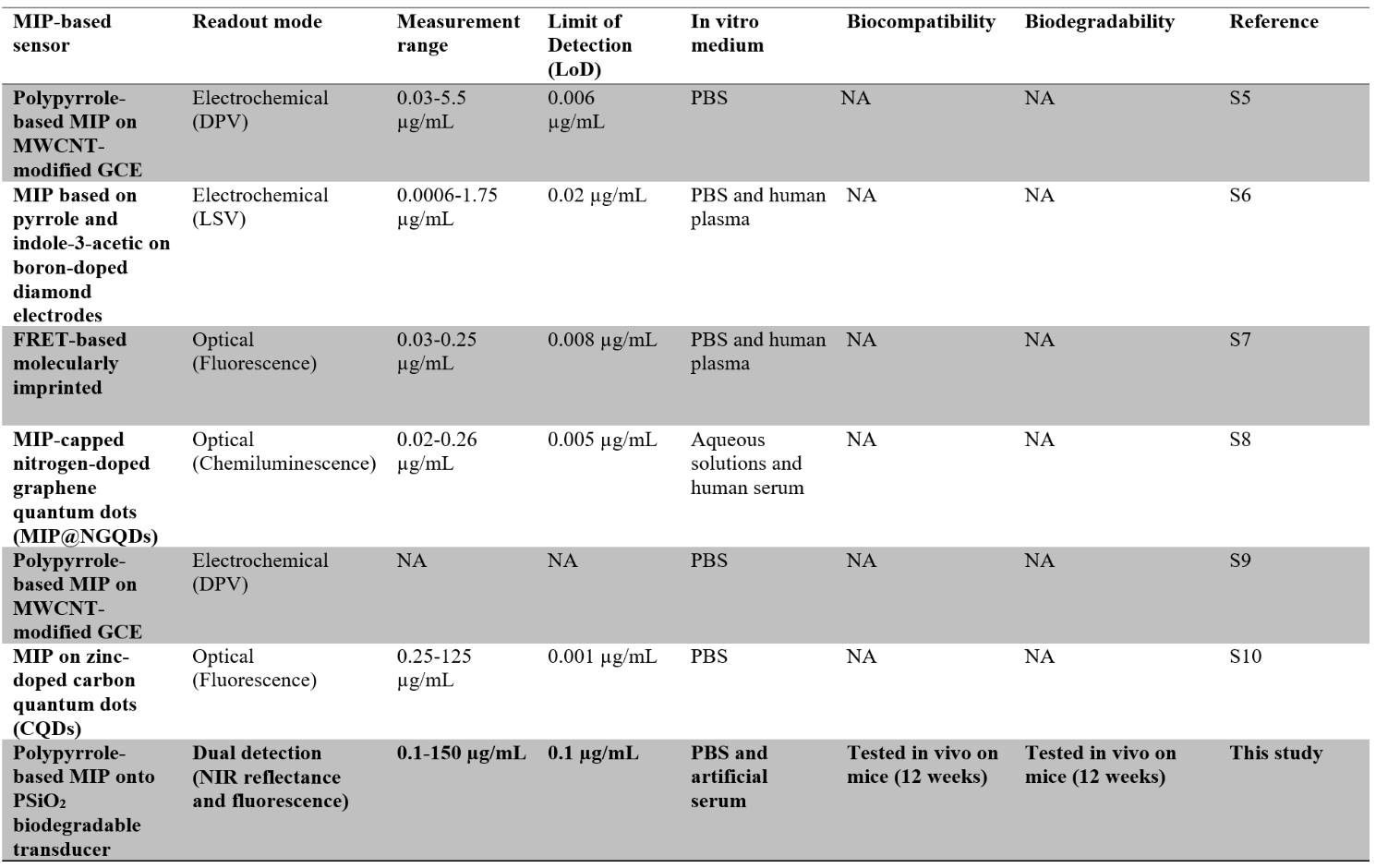
**

**Table S5.** Comparison of MIP-based sensors for doxorubicin. MWCNT = Multi-walled carbon nanotubes; GCE = Glassy Carbon Electrode; DPV = Differential Pulse Voltammetry; LSV = Linear Sweep Voltammetry; FRET = Fluorescence Resonance Energy Transfer; NA = Not Available.

**References**

S1. Arroyo-Currás, N., Somerson, J., Vieira, P. A., Ploense, K. L., Kippin, T. E. & Plaxco, K. W. Real-time measurement of small molecules directly in awake, ambulatory animals. *Proceedings of the National Academy of Sciences* **114**, 645–650 (2017).

S2. Harvey, J. D., Williams, R. M., Tully, K. M., Baker, H. A., Shamay, Y. & Heller, D. A. An *in Vivo* Nanosensor Measures Compartmental Doxorubicin Exposure. *Nano Lett.* **19**, 4343–4354 (2019).

S3. Seo, J.-W., Fu, K., Correa, S., Eisenstein, M., Appel, E. A. & Soh, H. T. Real-time monitoring of drug pharmacokinetics within tumor tissue in live animals. *Sci. Adv.* **8**, (2022).

S4. Corsi, M., Maurina, E., Surdo, S., Vandini, E., Daini, E., Vilella, A., Leo, G., Farshchian, M., Grisendi, G., Golinelli, G., Dominici, M., Bocci, G., Giuliani, D. & Barillaro, G. In vivo and in situ monitoring of doxorubicin pharmacokinetics with an implantable bioresorbable optical sensor. *Sci. Adv.* **11**, (2025).

S5. Soosani, Z., Rezaei, B., Heydari-Bafrooei, E. & Ensafi, A. A. Chemical Sensors Based on Molecularly Imprinted Polymers Can Determine Drug Release Kinetics from Nanocarriers without Filtration, Centrifugation, and Dialysis Steps. *ACS Sens.* **8**, 1891–1900 (2023).

S6. Ishii, K., Ogata, G., Yamamoto, T., Sun, S., Shiigi, H. & Einaga, Y. Designing Molecularly Imprinted Polymer-Modified Boron-Doped Diamond Electrodes for Highly Selective Electrochemical Drug Sensors. *ACS Sens.* **9**, 1611–1619 (2024).

S7. Xu, Z., Deng, P., Li, J., Xu, L. & Tang, S. Molecularly imprinted fluorescent probe based on FRET for selective and sensitive detection of doxorubicin. *Materials Science and Engineering: B* **218**, 31–39 (2017).

S8. Amjadi, M. & Jalili, R. Molecularly imprinted polymer-capped nitrogen-doped graphene quantum dots as a novel chemiluminescence sensor for selective and sensitive determination of doxorubicin. *RSC Adv.* **6**, 86736–86743 (2016).

S9. Dashtaki, R. M., Dashtaki, S. M., Heydari-Bafrooei, E. & Piran, M. J. Enhancing the Predictive Performance of Molecularly Imprinted Polymer-Based Electrochemical Sensors Using a Stacking Regressor Ensemble of Machine Learning Models. *ACS Sens.* **10**, 3123–3133 (2025).

S10. Zhang, H., Fang, F., Yi, J., Sun, Y. & Wang, X. Molecularly imprinted polymer coupled with zinc-doped carbon quantum dots as a fluorescent probe for the determination of doxorubicin hydrochloride. *New Journal of Chemistry* **49**, 3200–3206 (2025).
